# Withaferin A Increases the Effectiveness of Immune Checkpoint Blocker for the Treatment of Non-Small Cell Lung Cancer

**DOI:** 10.3390/cancers15123089

**Published:** 2023-06-07

**Authors:** Roukiah Khalil, Ryan J. Green, Kavya Sivakumar, Payal Varandani, Srinivas Bharadwaj, Shyam S. Mohapatra, Subhra Mohapatra

**Affiliations:** 1 Department of Molecular Medicine, Morsani College of Medicine, University of South Florida, Tampa, FL 33612, USA; 2 Department of Internal Medicine, Morsani College of Medicine, University of South Florida, Tampa, FL 33612, USA; 3 Taneja School of Pharmacy, University of South Florida, Tampa, FL 33612, USA; 4 Department of Veterans Affairs, James A. Haley Veterans Hospital, Tampa, FL 33612, USA

**Keywords:** PD-L1, immune checkpoint blockers, immunotherapy, Withaferin A, combination therapy, lung cancer

## Abstract

**Simple Summary:**

Lung cancer is the leading cause of cancer-related deaths. Immunotherapy activates the patient’s immune system to identify and kill cancer cells. Moreover, memory immune cells are formed that prevent the recurrence of cancer, leading to durable responses. However, only 20% of patients benefit from immunotherapy because the tumor-derived factors suppress the immune response. Herein, we tested if Withaferin A (a herbal compound) can make immunotherapy more effective in lung cancer patients. We found that Withaferin A induces the production of molecules from lung cancer cells that increase the infiltration of immune cells but are not able to kill cancer cells. Notably, in an immunocompetent mouse model of lung cancer, treatment with a combination of Withaferin A and an immunotherapy regimen showed more effectiveness than immunotherapy alone in activating immune cells and reducing tumor growth. This study presents a novel approach that can be tested clinically to improve lung cancer immunotherapy.

**Abstract:**

Treatment of late-stage lung cancers remains challenging with a five-year survival rate of 8%. Immune checkpoint blockers (ICBs) revolutionized the treatment of non-small cell lung cancer (NSCLC) by reactivating anti-tumor immunity. Despite achieving durable responses, ICBs are effective in only 20% of patients due to immune resistance. Therefore, synergistic combinatorial approaches that overcome immune resistance are currently under investigation. Herein, we studied the immunomodulatory role of Withaferin A (WFA)—a herbal compound—and its effectiveness in combination with an ICB for the treatment of NSCLC. Our in vitro results show that WFA induces immunogenic cell death (ICD) in NSCLC cell lines and increases expression of the programmed death ligand-1 (PD-L1). The administration of N-acetyl cysteine (NAC), a reactive oxygen species (ROS) scavenger, abrogated WFA-induced ICD and PD-L1 upregulation, suggesting the involvement of ROS in this process. Further, we found that a combination of WFA and α-PD-L1 significantly reduced tumor growth in an immunocompetent tumor model. Our results showed that WFA increases CD-8 T-cells and reduces immunosuppressive cells infiltrating the tumor microenvironment. Administration of NAC partially inhibited the anti-tumor response of the combination regimen. In conclusion, our results demonstrate that WFA sensitizes NSCLC to α-PD-L1 in part via activation of ROS.

## 1. Introduction

Lung cancer is the leading cause of cancer deaths with 127,070 estimated deaths in 2023 in the USA [1]. Despite the abundance of anti-cancer treatments, the five-year survival rate can be as low as 8% in late-stage lung cancer [2]. The role of the immune system in cancer prevention by immunosurveillance was harnessed for the development of immunotherapies including immune checkpoint blockers (ICBs) [3]. Cancer immunosurveillance is achieved when antigen presenting cells (APCs) process and present tumor-associated antigens to elicit a cytotoxic T-cell (CTL) response [4]. Consequently, two signals are required for T-cell activation: an antigen-major histocompatibility complex-I (MHC-I) and T-cell receptor (TCR) interaction, and a second-the T-cell co-stimulatory molecule CD-28 and its tumor-expressed cognate ligand B7-1. Following activation, CTLs release apoptotic mediators (e.g., perforin, granzyme B), inducing tumor cell death. To evade immune detection, tumors upregulate the expression of inhibitory immune checkpoint (IC) molecules to counteract T-cell co-stimulatory signals, therefore inactivating CTLs [5]. For example, cytotoxic T-lymphocyte protein 4 (CTLA4) is an inhibitory IC molecule that competitively binds B7-1 and therefore inhibits its interaction with CD28. Another IC molecule of significance is the programmed death protein-1 (PD-1) which inhibits T-cell signaling and activation by interacting with its ligand PD-L1 expressed on tumor cells. ICBs are monoclonal antibodies that inhibit the interaction of IC molecules and their ligands, thus restoring T-cell activation. Based on several clinical trials comparing the effectiveness of ICBs to first- or second-line chemotherapies, ICBs were FDA-approved and have been used for lung cancer treatment since 2015 [6]. One advantage of ICBs over other therapies is their ability to activate immunologic memory leading to a long-lasting response by eliminating recurring tumor cells [7]. However, only 17–20% of treated patients attain a durable response due to tumor-induced immunosuppression [8].

Tumor immunoresistance/immunosuppression can be broadly classified into tumor-intrinsic (tumor cell mediated) or tumor extrinsic resistance (host factors, microbiome, tumor microenvironment (TME)) [9]. Tumor intrinsic resistance involves poor immunogenicity, defective interferon signaling, and the upregulation of inhibitory IC molecules, while tumor-extrinsic resistance includes lack of immune infiltration or immunosuppressive cell infiltration (e.g., regulatory T-cells (T-regs) and myeloid-derived suppressor cells (MDSCs)) [10]. Therefore, certain factors/biomarkers are associated with a better response to ICB therapy [11]. For instance, non-small cell lung cancer (NSCLC) patients having a higher expression of the IC molecule programmed death ligand 1 (PD-L1) show a response to ICBs [12]. Furthermore, patients with higher tumor-infiltrating lymphocytes (immunologically hot) have a better response than tumors that lack immune cell infiltration (immunologically cold) [13]. Other predictors of patient response include microsatellite instability and tumor mutational burden [14]. Therefore, biomarkers such as higher PD-L1 expression levels and tumor immune infiltration are associated with better response and are used to assess whether a patient is a good candidate for ICB therapy.

To overcome the immune resistance and improve patient response to ICBs, current trials attempt to combine ICBs with drugs/radiation that target the immunosuppressive mechanisms in the TME [15]. For example, anthracyclines and oncolytic viruses induce immunogenic cell death (ICD) in which dying tumor cells release or express danger-associated molecular patterns (DAMPs) such as calreticulin (CRT), High Mobility Group Box-1 (HMGB-1), and heat shock proteins (HSP-70 and HSP-90) [16]. Consequently, APCs detect DAMPs as ‘eat-me signals’ and engulf dying tumor cells, to activate an anti-tumor CTL response. As a result, ICD can convert an immunologically cold into an immunologically hot TME [17]. Furthermore, radiotherapy and some chemotherapeutics (e.g., mitomycin C and cisplatin) that increase PD-L1 expression on tumor cells sensitize lung cancer to α-PD-L1 therapy [15,18,19]. However, many of these combinations are not clinically applicable due to the additive side effects of the individual agents including cardiotoxicity [20,21]. Therefore, there is still an unsolved quest to find therapies that expand the use of ICBs to a wider patient population by overcoming immune resistance without additive toxicity.

Withaferin A (WFA) is a broad-spectrum anticancer steroidal lactone isolated from the leaves and roots of Withania Somnifera (WS) [22]. Mechanistically, WFA induces apoptotic and ferroptotic cell death, and inhibits angiogenesis, metastasis, and cancer stemness [23,24,25]. Additionally, WFA exerts its anti-cancer effects by targeting several downstream molecules. For instance, WFA was found to activate Liver-x-receptor signaling and inhibit NF-κB, VEGF, MMPs, cyclins, CDKs, and EGFR [26,27,28,29]. One important mechanism of WFA-induced cytotoxicity in cancer cells is mitochondrial dysfunction and subsequent increased oxidative stress and reactive oxygen species (ROS) production [30]. Specifically, WFA was shown to inhibit complex III function and disturb the electron transport chain leading to increased ROS production [31]. Although WFA is toxic to cancer cells, studies show that it can protect normal non-cancerous tissues including lung fibroblasts, neurons, and cardiomyocytes from injury. For instance, WFA was found to protect cardiomyocyte cells from oxidative damage by activating several antioxidant pathways including SOD-2 and SOD-3 [32]. Moreover, normal cell such as WI-38 lung fibroblasts and PBMCs remain viable at WFA concentrations that were toxic to A549 NSCLC cells [33]. This indicates that WFA can play a dual role as a pro-oxidant or an antioxidant agent based on the cellular context. Although WS was found to be an immunostimulant, only a few studies show the effect of WFA on anti-tumor immunity. In BALB/c mice, WS increases B- and T-cell proliferation as well as T-helper 1 response [34]. Moreover, a clinical study shows that WS extract increases TBNK and IgG levels in healthy subjects [35]. In a breast cancer immunocompetent mouse model, WFA was shown to target the immunosuppressive MDSC population by reducing their tumor infiltration and immunosuppressive function [36].

Despite being well studied, the immunomodulatory properties of WFA and its role in modulating the effectiveness of ICB in NSCLC remain unexplored. We reasoned that WFA treatment can overcome tumor immune resistance and sensitize NSCLC to ICB therapy. To test this idea, we first investigated whether WFA could activate anti-tumor immunity by inducing ICD or/and altering IC molecule expression. We then tested the effectiveness of the combination treatment of WFA and α-PD-L1 as an ICB in an NSCLC immunocompetent mouse model. Our in vitro findings show that WFA-induced apoptotic ICD is associated with the DAMPs from the dying cells. Additionally, WFA altered the expression of IC molecules in NSCLC cell lines which sensitizes cancer cells to α-PD-L1 therapy. Importantly, we show for the first time that WFA sensitized an ICB-resistant tumor mouse model to α-PD-L1 therapy and restored anti-tumor immunity by altering tumor-immune infiltration and ROS levels in the TME in vivo.

## 2. Materials and Methods

### 2.1. Chemotherapeutic Drugs and Antibodies

WFA powder (5119-48-2) was purchased from ChromaDex Standards (Los Angeles, CA, USA) and reconstituted using Dimethyl Sulfoxide (DMSO) (Sigma-Aldrich, Burlington, MA, USA) to form 100 and 10 mM stock solutions, and stored at −20 °C in a freezer before use. For in vitro cell treatments, WFA stock solutions were diluted using complete culture media at the desired concentration (ranging from 10 µM to 0.4 µM). For in vivo treatments, WFA was diluted using Glyceryl Trioctanoate ((Sigma-Aldrich, Burlington, MA, USA) at the desired dose (4 mg/kg). In vivo mouse α-PD-L1 antibody (clone 10F.9G2) and rat IgG2b isotype control (clone LTF-2) were purchased from BioXcell (Lebanon, NH, USA). N-acetyl cysteine (NAC) was purchased from Sigma-Aldrich (Burlington, MA, USA) (A9165-25G) and L-Glutathione was purchased from Spectrum mfg. Corp (Gardena, CA, USA) (39G505). NAC or Glutathione were solubilized in PBS to make a 0.5 M solution and freshly prepared before each experiment. STATTIC (STAT3 inhibitor), PX-478 (HIF1α inhibitor), and Brusatol (NRF-2 inhibitor) were all purchased from Selleck Chemicals (Houston, TX, USA) and solubilized in DMSO to create 10 mM stocks.

### 2.2. Cell Lines

Human cell lines H1650, A549, HCT-116, MDA-MB-231 and mouse cell lines Lewis Lung Carcinoma (LLC) and 4T1 were purchased from the American Type Culture Collection (ATCC). The murine cell line MC-38 was provided by Dr. Shari Pilon-Thomas (Moffitt Cancer Center). H1650, A549, 4T1, and MDA-MB-231 cells were cultured in Roswell Park Memorial Institute (RPMI) media (Cytiva HyClone, Logan, UT, USA) supplemented with 10% heat inactivated fetal bovine serum (FBS) (Thermo Fisher Scientific, Waltham, MA, USA, 900-108) and 1% anti-bacterial/anti-mycotic solution (Cytiva HyClone, Logan, UT, USA, SV3007901). LLC cells were cultured in Dulbecco’s Modified Eagle Medium media (Gibco, Grand Island, NY, USA) supplemented with 10% heat inactivated FBS and 1% anti-bacterial/anti-mycotic solution. The colorectal cancer cell line MC-38 was cultured in RPMI media supplemented with 2 mM L-glutamine (Gibco, Grand Island, NY, USA), 0.1 mM nonessential amino acids (Gibco, Grand Island, NY, USA), 1 mM sodium pyruvate (Gibco, Grand Island, NY, USA), 100 U/mL penicillin, 100 mg/mL streptomycin, and 10% heat inactivated FBS. The human colorectal cancer cell line HCT-116 was cultured in McCoy’s 5A medium (Gibco, Grand Island, NY, USA) supplemented with 10% heat inactivated FBS and 1% anti-bacterial/anti-mycotic solution. Bone marrow-derived dendritic cells were cultured in complete RPMI media supplemented with 2 mM Glutamax (Gibco, Grand Island, NY, USA)), 50 µM β-mercaptoethanol, (Fisher Scientific, Waltham, MA, USA), and 20 ng of granulocyte monocyte colony stimulating factor (GM-CSF) (Pepro Tech, Cranbury, NJ, USA, 315-03) per ml. All cell lines were incubated at 37 °C in a humidified 5% CO_2_ incubator.

### 2.3. Cell Titer-Glo^®^ Assay

LLC, H1650, or A549 was plated in 96-well plates and treated with WFA (serial dilutions 10–0.078 µM) in triplicates. DMSO control was used to ensure that the observed cell death is due to WFA treatment only. Forty-eight hours after treatment, cell viability was determined using Cell Titer-Glo^®^ assay (Promega^®^, Madison, WI, USA) according to the manufacturer’s instructions. Luminescence was measured using a white well plate in a Bio-Tek Synergy H4 plate reader (Bio Tek, Winooski, VT, USA). Average luminescence normalized to untreated control cells was graphed against log WFA concentration and non-linear regression was performed to determine the inhibitory concentration 50 (IC50) value of WFA in each cell line.

### 2.4. Annexin V Assay

NSCLC cell lines were treated with different concentrations of WFA for forty-eight hours then collected using Accutase^®^ cell detachment solution (Innovative Cell Technologies, San Diego, CA, USA). Apoptotic cell death was measured by flow cytometry using Propidium iodide or 4′,6-diamidino-2-phenylindole (DAPI) viability dye and eBioscience™ Annexin V Apoptosis Detection Kit Allophycocyanin (APC) (Thermo Fisher Scientific, Waltham, MA, USA) according to the manufacturer’s protocol.

### 2.5. Flow Cytometry

In vitro WFA-treated or control cells were collected using Accutase^®^ cell detachment solution and stained with FACS-diluted (PBS, 10% FBS, 2 mM EDTA) pre-titrated fluorophore-conjugated or primary unconjugated antibody solutions (Supplemental Table S1) for 30 min on ice in the dark. In case of fluorophore-conjugated antibodies, samples are washed and immediately acquired after adding DAPI viability dye, while samples stained with unconjugated antibodies are incubated with the proper secondary fluorophore-conjugated antibody for 30 min before washing and acquisition. For in vivo experiments, the processed tumor or spleen single cell suspensions were used for surface staining with the pre-titrated antibodies (Supplemental Table S1) for 30 min on ice in the dark. To exclude the dead cells from the analysis, a fixable live/dead Zombie Aqua viability kit (Biolegend, San Diego, CA, USA) was used according to the manufacturer’s protocol. Samples were then fixed using 2% PFA for 15 min then washed and stored at 4 °C before acquisition. Samples were acquired using a Becton Dickenson Biosciences (BD, Franklin Lakes, NJ, USA) FACS Canto II or LSR II system at the University of South Florida COM Fred Wright Jr Flow Cytometry Core. Analysis was performed using FlowJo 8.7 software (BD, Franklin Lakes, NJ, USA).

### 2.6. HMGB-1 Assay

LLC cells were plated and treated with WFA or doxorubicin (ICD inducer) for 48 h. Supernatants were collected and centrifuged at 1000 RPM for 10 min. The levels of secreted HMGB-1 were measured using the HMGB-1 detection ELISA kit (Chondrex, 6010, Woodinville, WA, USA) according to the manufacturer’s protocol.

### 2.7. Ex Vivo Dendritic Cell Activation Assay

Myeloid progenitor cells were isolated from the femurs of C57BL/6 mice as described previously [37]. The isolated cells were cultured in RMPI media supplemented with GM-CSF and refed as necessary. On day 8, the differentiated DCs (loosely attached) were collected, counted, and added to WFA-pretreated LLCs or untreated control LLCs at a ratio of 5:1 of DC: LLC cells. Twenty-four hours post co-culture, the DC were collected and stained with anti-CD11c, anti-CD80, anti-CD86, and anti-MHCII antibodies (BioLegend, San Diego, CA, USA). Then the samples were acquired using a BD LSR II flow cytometer and the data were analyzed using FlowJo 8.7 software (BD, Franklin Lakes, NJ, USA).

### 2.8. Reverse Transcription and Quantitative PCR

Total cellular RNA was isolated from collected cell pellets using TRIzol reagent (Invitrogen, Waltham, MA, USA) and then quantified using the 2000 Nanodrop spectrophotometer (Thermo Fisher Scientific, Waltham, MA, USA). cDNA synthesis was performed using the Verso cDNA synthesis kit (Thermo Fisher Scientific, Waltham, MA, USA). qPCR was then performed using Forget-Me-Not™ EvaGreen^®^ qPCR Master Mix (Biotium, Fremont, CA, USA) in a Bio-Rad CFX384 thermocycler according to the manufacturer’s instructions. The CFX Maestro software version 2.3 (Bio-Rad, Hercules, CA, USA) was used to calculate the ∆Ct and ∆∆Ct values and gene expression was normalized to housekeeping genes glyceraldehyde 3-phosphate dehydrogenase (GAPDH) or β-actin. The sequences of PCR primers used are listed in Supplemental Table S2 (Integrated DNA Technologies, Inc, Coralville, IA, USA).

### 2.9. Protein Extraction and Western Blot

Total protein was isolated using Radioimmunoprecipitation Assay (RIPA) buffer (Thermo Fisher Scientific, Waltham, MA, USA) and sonication of cell pellets using Branson digital sonifier 450. The lysates were centrifuged at 16,000 rpm for 20 min and the supernatants containing total cellular proteins were collected. Then, protein levels were quantified using a Pierce Coomassie Protein assay kit (Thermo Fisher Scientific, Waltham, MA, USA) according to the manufacturer’s instruction and the absorbance values at 595 nm were measured using a plate reader (Bio Tek, Winooski, VT, USA) and used to calculate protein concentrations. Fifty micrograms of protein were resolved by SDS-polyacrylamide gel electrophoresis (Bio-Rad, Hercules, CA, USA) and transferred to a 0.2 µm nitrocellulose membrane (Bio-Rad, Hercules, CA, USA) at 80 V for 2 h. Blots were blocked using 5% milk in TBS-T or 5% bovine albumin serum in the case of phosphoproteins for 1 h at room temperature. Blots were then incubated with the primary antibody (concentration 1:1000) in the blocking solution at 4 °C overnight with gentle agitation. Blots were washed with TBS-T three times and incubated with the secondary HRP-conjugated antibodies for an hour at room temperature. Primary and secondary antibodies used for western blotting are listed in the Supplemental Table S3. Protein bands were detected using SuperSignal™ West Pico PLUS Chemiluminescent Substrate or West Femto Maximum Sensitivity Substrate (Thermo Fisher Scientific, Waltham, MA, USA) and imaged using the ChemiDoc XRS ™ imaging system (Bio-Rad, Hercules, CA, USA) according to the manufacturer’s instructions. Band density was measured using Image J software version 1.54d and was normalized to housekeeping proteins (β-actin or Vinculin). Protein levels in the treatment groups were normalized to the untreated control and an average of 2 independent experiments ± SEM are shown in the bar graphs.

### 2.10. Ingenuity Pathway Analysis (IPA)

To identify the downstream regulators that contribute to WFA-induced PD-L1 upregulation, we constructed a gene interaction network between the PD-L1 signaling pathway and ROS (nitric oxide, oxygen radical, hydrogen peroxide, and lipoxygenase) signaling using the known molecular connections obtained from the IPA database (Qiagen, Redwood City, CA, USA). Fold change data from MCF-7 breast cancer cells treated with WFA (700 nM for 72 h) were obtained from the NCBI Gene Omnibus Expression (GEO) database (Series GSE53049) and overlaid onto the network to yield predictions of activation/inhibition in the IPA software version 90348151 (Qiagen, Redwood City, CA, USA).

### 2.11. SiRNA Transfection

Cells were transfected using the TransIT-TKO^®^ transfection kit (Mirus Bio, Madison, WI, USA) according to the manufacturer’s instructions. Briefly, LLC or H1650 cells were seeded in 24-well plates and incubated for 24 h. The transfection reagent was prepared by mixing NRF-2 or non-targeting siRNA with TransIT-X2^®^ reagent in antibiotic-free, serum-free Opti-MEM^®^ (Gibco, Grand Island, NY, USA) medium to reach a final concentration of 25 nM. The mixture was added to the cells for twenty-four hours to allow transfection. Subsequently, the cells were treated with WFA and incubated for twenty-four hours before collection. Non-targeting siRNA, mouse and human NRF-2 siRNA SMART pools were purchased from Dharmacon™ Reagents (Lafayette, CO, USA, D-001810-01-20, L-003755-00-0010 and L-040766-00-0010).

### 2.12. Animal Studies

C57BL/6 mice were purchased from the Jackson Laboratory (Bar Harbor, ME, USA) and acclimated for a week in the University of South Florida comparative medicine facility at the Morsani College of Medicine. To establish the LLC syngeneic tumors, C57BL/6 mice were injected with a half million cells on the right flank and monitored until tumors were first palpable (2–3 mm diameter). Mice were then randomized into treatment groups (n = 5 per group), to receive either vehicle (10% DMSO, 90% Glyceryl Trioctanoate), WFA (4 mg/kg), α-PD-L1(200 µg/mouse), isotype antibody (200 µg/mouse), NAC (100 mg/kg), a combination of α-PD-L1 and WFA, or a combination of WFA+ α-PD-L1 and NAC intraperitonially. Treatments were administered every other day with a total of five treatments and mice body weights and tumor volumes were monitored using a caliper (Fisher Scientific, Waltham, MA, USA, 15-077-957). Tumor volumes (mm^3^) were calculated using the equation (length^2^ × width/2), where the length is defined as the bigger tumor dimension. When the control tumors reached 10 mm x10 mm, mice were euthanized using CO_2_ euthanasia; then, tumors and spleen were collected for downstream analyses. Briefly, spleens and tumors were collected and dissociated mechanically or using the Miltenyi Biotec mouse tumor dissociation kit (130-096-730) and filtered through 70 µm strains (Thermo Fisher Scientific, Waltham, MA, USA). Red blood cells were lysed using the ACK lysing buffer (Thermo Fisher Scientific, Waltham, MA, USA). Single cell suspensions were then used for flow cytometry. All protocols were approved by USF institutional animal care and use committee.

### 2.13. Statistical Analysis

Each experiment was repeated at least twice, and the results are represented as mean ± the standard error of the mean, SEM. To calculate statistical significance, an unpaired Student’s *t*-test was used when comparing two groups, while Analysis of Variance (ANOVA) was used when comparing multiple groups. The Fisher LSD post hoc test was performed to compare the means of the treatment groups to that of the control group. Analyses were carried out and graphs were plotted using GraphPad Prism 8.0.1. * or # *p* < 0.05, ** or ## *p* < 0.01, *** or ### *p* < 0.001, **** *p* < 0.0001.

## 3. Results

### 3.1. WFA Induces ER-Stress Mediated Apoptosis in NSCLC

**Figure 1:**
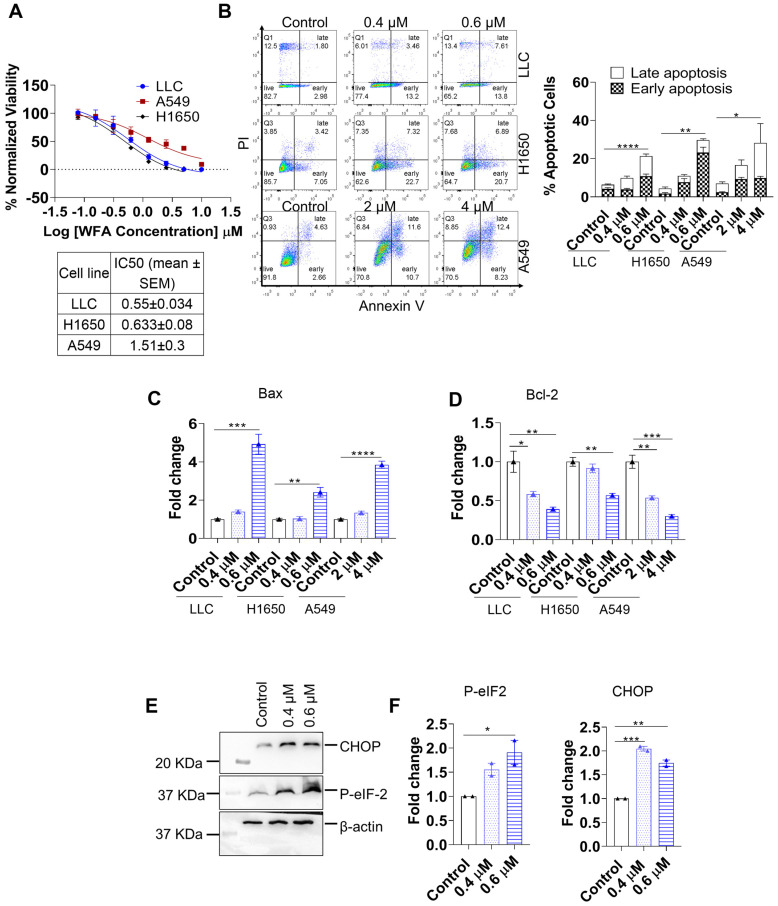
WFA induces ER stress-mediated apoptosis in NSCLC cell lines. (**A**) NSCLC cells were plated on 96-well plates and treated with different concentrations of WFA (10–0.1 μM) in triplicates. Forty-eight hours post-treatment, cell viability was determined using Cell Titer-Glo assay. Luminescence values were normalized to control untreated cells and IC50 values are represented as the mean of two independent experiments ± SEM. (**B**) NSCLC cells were treated with WFA for forty-eight hours, then collected to measure apoptotic cell death using Annexin V assay in LLC cells, H1650 cells, and A549 cells. Percentages of early (annexin^+^DAPI^−^) and late (annexin^+^DAPI^+^) apoptotic cells are represented as means of two separate experiments ± SEM and statistical significance was calculated using one-way ANOVA and a Fisher LSD post hoc test was used to compare the mean of each group to that of the control group. (**C**,**D**) WFA treated cells were collected in Trizol and total RNA was isolated to measure the levels of Bax (**C**) and Bcl2 (**D**) mRNA using qRT-PCR. PCR experiments were repeated at least twice, and one representative experiment is shown as mean ± SEM of the technical replicates. (**E**,**F**) Western blot of ER stress markers p-eIF-2 and CHOP was performed using total lysates of WFA-treated LLC cells (**E**). The quantification of western blot band density for p-eIF-2 and CHOP (**F**) performed using Image J software v.1.54d and normalized to housekeeping control protein in LLC cells (β-actin) shown. The mean fold change in band density from the control is shown in the bar graphs ±SEM and one-way ANOVA and a Fisher LSD post hoc test were used to calculate statistical significance. * *p* < 0.05, ** *p* < 0.01, *** *p* < 0.001, **** *p* < 0.0001. Data were obtained from two independent experiments. The original western blot figures could be found in File S1I.

Despite being originally considered non-immunogenic, recent findings show that certain apoptotic triggers can induce ICD. For example, stressors that induce prolonged ER stress and unfolded protein response can lead to immunogenic apoptotic cell death [38]. Previously, WFA was shown to induce apoptotic cell death in NSCLC cell lines [33]. However, it is unclear whether it can activate the ER stress pathway and ICD. To confirm the mechanism by which WFA targets NSCLC, we treated the cell lines (LLC, H1650 and A549) with different concentrations of WFA for forty-eight hours. Cell Titer-Glo assay showed that WFA was toxic in these cell lines with IC50 values ranging from 0.5 to 1.5 µM (Figure 1A). Consequently, we performed an annexin V assay that showed a significant increase in early and late apoptotic cell populations in WFA-treated cells compared to their untreated counterparts (Figure 1B). To investigate the molecular mechanisms of WFA-induced apoptosis, we measured the changes in both pro and anti-apoptotic marker transcripts using qRT-PCR and Western blotting. Our PCR studies revealed that WFA increased the transcription of the pro-apoptotic Bax protein transcripts (Figure 1C), Bak-1 (Figure S1A), and BAD (Figure S1D). Moreover, WFA treatment reduced the transcripts of the anti-apoptotic proteins Bcl-2 (Figure 1D), Xiap, and Survivin (Figure S1B,C,E,F). Moreover, western blotting showed that WFA increased PARP cleavage in LLC and H1650 cells and reduced the levels of BCL-XL in H1650 cells (Figure S1G,H). We then investigated whether WFA induces ER stress in NSCLC cell lines by quantifying the levels of ER stress markers using western blotting. We found that WFA increases the levels of C/EBP homologous protein (CHOP) and the phosphorylation of the eukaryotic initiation factor eIF-2 (Figure 1E,F). In summary, we identified that WFA induces ER-stress mediated apoptosis in NSCLC cell lines.

### 3.2. WFA Induces ICD in NSCLC Cell Lines

**Figure 2:**
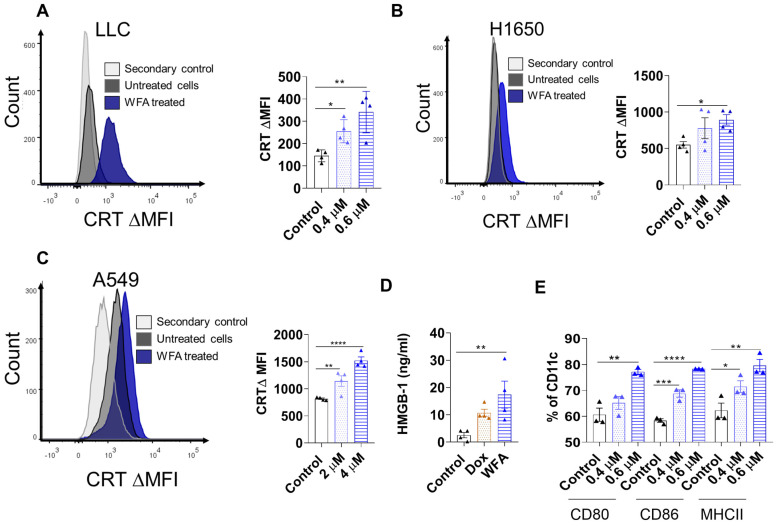
WFA induces ICD in NSCLC cell lines. Cells were treated with WFA for 48 h then collected using Accutase cell detachment solution. (**A**–**C**) CRT levels were determined by flow cytometry in LLC (**A**), H1650 (**B**), and A549 (**C**) cells. (**D**) The levels of secreted HMGB-1 in LLC cell treated with 0.6 µM WFA or 30 nM doxorubicin were measured by ELISA and the means of two independent experiments ± SEM are represented in the bar graph. (**E**) Bone marrow-derived DC were co-cultured with WFA-pretreated LLC cells (24 h) in a ratio 5:1 of DC:LLC cells for 24 h. DC activation markers (CD80, CD86, and MHC-II) were examined by flow cytometry. The means of at least 2 independent experiments ± SEM is represented, and one-way ANOVA was used to calculate statistical significance. * *p* < 0.05, ** *p* < 0.01, *** *p* < 0.001, and **** *p* < 0.0001.

ICD is characterized by the expression or release of DAMPs such as CRT and HMGB-1 that act as a (eat me) signal to activate APCs [39]. Consequently, active APC can present tumor antigens along with the stimulatory signals essential to produce an anti-tumor T-cell response [40]. To determine if WFA can induce ICD in lung cancer, we treated LLC, H1650 and A549 cells with WFA and then collected the cells or supernatants to measure the levels of cell surface expressed CRT (ecto-CRT) or secreted HMGB-1, respectively. We found that WFA treatment increased ecto-CRT expression in LLC, H1650 cells and A549 cells (Figure 2A–C) by flow cytometry analysis. These findings prompted us to test if WFA can also induce ICD in other types of cancer (e.g., colorectal cancer). To test this hypothesis, we treated the murine MC38 and human HCT-116 colorectal cancer cell lines with WFA and then measured the change in ecto-CRT levels using flow cytometry. Interestingly, WFA increased the levels of ecto-CRT in both MC38 and HCT-116 cell lines (Figure S2A,B). To measure the levels of secreted HMGB-1, we collected the supernatants of WFA-treated LLC cells and centrifuged them to remove any dead cells or debris. Using the HMGB-1 ELISA kit, we found that WFA treatment increased the HMGB-1 levels secreted in the supernatants of WFA-treated LLC cells (Figure 2D). To confirm that the release of DAMPs by WFA treated cells can activate APCs, we isolated bone marrow-derived myeloid progenitors from C57BL/6 mice and allowed their differentiation into DCs (day 8). We then collected the bone marrow-derived DCs and co-cultured them with WFA-pretreated LLC for twenty-four hours. Subsequently, DCs were collected and their expression of the activation markers CD80, CD86, and MHC-II was measured using flow cytometry (Figure S3). Our results indicate that DCs co-cultured with WFA-treated cells expressed higher levels of the activation markers CD80, CD86, and MHC-II than those cultured with untreated controls (Figure 2E). Moreover, qRT-PCR shows that WFA increased IFN-α, characteristic of ICD in LLC and H1650 cells [41] (Figure S4A,B). Our findings confirm that WFA can induce ICD in NSCLC cells and colorectal cancer cells by promoting the release/expression of DAMPs which consequently activate APC.

### 3.3. WFA Increases PD-L1 Expression in NSCLC Cell Lines

**Figure 3:**
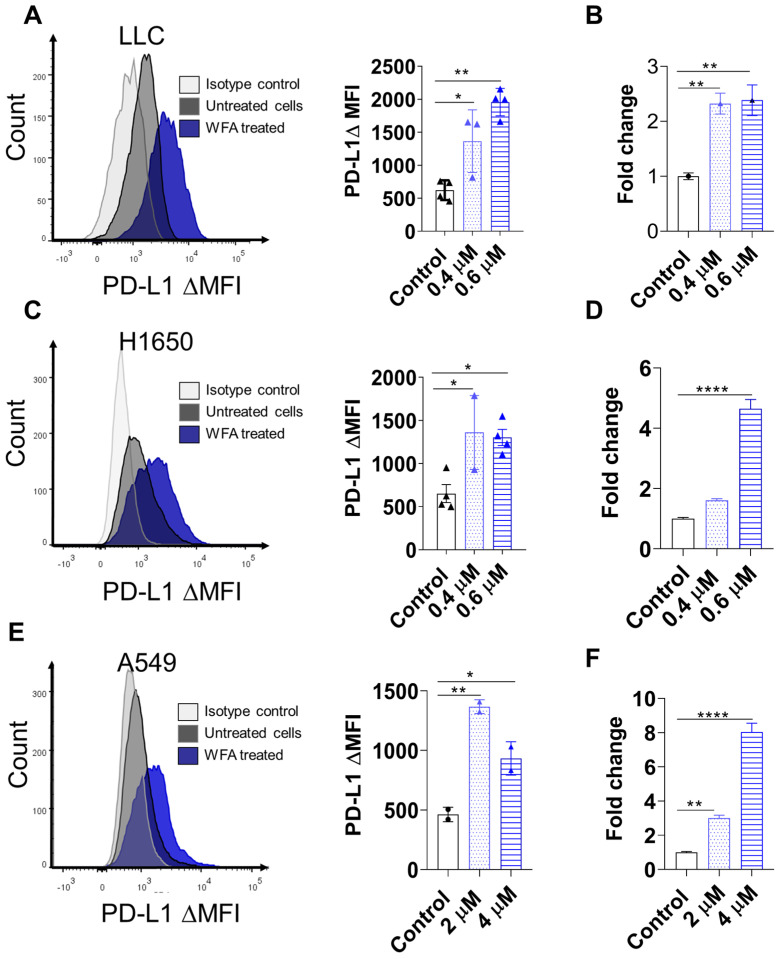
WFA upregulates PD-L1 surface expression in NSCLC cell lines. Cells were treated with WFA for 48 h. (**A**,**C**,**E**) PD-L1 surface expression determined using flow cytometry LLC (**A**), H1650 (**C**), and A549 (**E**). (**B**,**D**,**F**) PD-L1 expression was measured using qRT-PCR LLC (**B**), H1650 (**D**), A549 (**F**). PCR experiments were repeated twice and the mean ± SEM of the technical replicates are represented. For the rest, the means of two independent experiments ± SEM are shown, and statistical significance was calculated using one-way ANOVA and a Fisher LSD post hoc test. * *p* < 0.05, ** *p* < 0.01, and **** *p* < 0.0001.

Due to the important role of PD-L1 as a biomarker of response to ICB therapy, we investigated the effect of WFA on PD-L1 expression in NSCLC cell lines. Towards this goal, we collected WFA-treated NSCLC cell lines to measure PD-L1 surface expression using flow cytometry. We found that WFA treatment consistently increased PD-L1 expression in LLC, H1650, and A549 cell lines (Figure 3A,C,E). Consistently, qRT-PCR (Figure 3B,D,F) and western blotting (Figure S2E–G) confirmed that WFA induced PD-L1 expression in NSCLC cell lines. We then investigated whether WFA can also increase PD-L1 expression in other types of cancers. Similarly, WFA increased PD-L1 expression in MC38, HCT116 colon cancer cell lines (Figure S2C,D). In conclusion, WFA upregulated PD-L1 expression in NSCLC and colorectal cancer cell lines which can sensitize these tumors to ICBs.

### 3.4. WFA Mediates PD-L1 Upregulation and ICD by Increasing ROS Production

**Figure 4:**
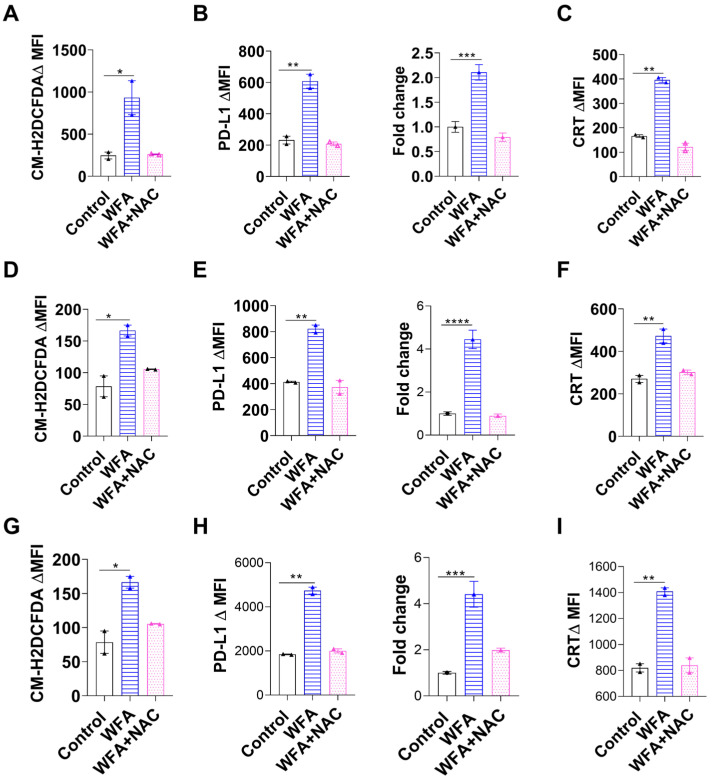
WFA-mediated ROS production is essential for PD-L1 upregulation and can be abrogated using NAC. NSCLC cell lines were treated with WFA (0.6 µM in LLC, H1650 cells, 4 µM in A549 cells), or a combination of WFA+5 mM NAC for 24 h. (**A**,**D**,**G**) The change in ROS levels were measured in LLC (**A**), H1650 (**D**), and A549 (**G**). (**B**,**E**,**H**) The change in PD-L1 levels was measured in LLC (**B**), H1650 (**E**), and A549 (**H**) cell lines using flow cytometry and qRT-PCR. (**C**,**F**,**I**) Ecto-CRT levels were measured in LLC (**C**), H1650 (**F**), and A549 (**I**) by flow cytometry. Means of two independent experiments ± SEM are shown, except for the PCR experiments that were repeated twice and for which one representative experiment is shown. Statistical significance was calculated using one-way ANOVA and a Fisher LSD post hoc test. * *p* < 0.05, ** *p* < 0.01, and *** *p* < 0.001, and **** *p* < 0.0001.

As our findings show that WFA induces PD-L1 upregulation and ICD in NSCLC, and colon cancer cell lines, we investigated the mechanism responsible for WFA-mediated PD-L1 expression changes. Others have shown that WFA induces cancer cell death mainly through increased ROS production which can be abrogated by ROS scavengers (e.g., NAC or Glutathione) [33,42]. Due to the central role of ROS in WFA-mediated cytotoxicity, we investigated if ROS production plays a role in PD-L1 upregulation as well. To test our hypothesis, we treated NSCLC cell lines with WFA, NAC, or a combination of both. To confirm the changes in ROS levels with each treatment, we stained these cells with ROS indicator CM-H2DCFDA and found that WFA increased ROS levels while NAC reduced them (Figure 4A,D,G). Interestingly, NAC treatment completely reversed the WFA-mediated PD-L1 increase as measured by both flow cytometry and qRT-PCR (Figure 4B,E,H). Moreover, NAC completely reversed ecto-CRT expression in NSCLC cell lines (Figure 4C,F,I). To confirm, we used Glutathione (GSH) as a ROS scavenger and found that it abrogated PD-L1 expression as well (Figure S4C–E). In addition, we examined the effects of NAC in WFA treated colorectal and breast cancer cell lines. Similar to NSCLC cell lines, we found that NAC completely reversed PD-L1 upregulation in MC-38, HCT-116, 4T-1, and MDA-MB-231 cell lines (Figure S4F–I, respectively). To investigate the downstream regulators that may be involved in ROS-mediated PD-L1 upregulation, we used IPA analysis of a publicly available dataset of WFA-treated MCF-7 breast cancer cells (Series GSE53049). First, we constructed a network showing the molecular connection between PD-L1 and ROS (nitric oxide, oxygen radical, hydrogen peroxide, and lipoxygenase). Subsequently, we overlaid the gene expression data obtained from the dataset onto the molecular network to predict the pathways involved in WFA-mediated PD-L1 upregulation (Figure S5). IPA predictions show that a complex network of multiple downstream regulators may be involved in PD-L1 upregulation. To validate the IPA results, we treated NSCLC cells with WFA or a combination of WFA and STATTIC (STAT-3 inhibitor), PX-478 (HIF1α inhibitor), or brusatol (NRF-2 inhibitor). Flow cytometry showed that neither STATTIC nor PX-478 were able to reverse WFA-induced PD-L1 upregulation (Figure S7A–C). However, adding brusatol—an NRF-2 inhibitor—to WFA treated cells completely abrogated the upregulation of PD-L1 in LLC, H1650, and A549 cells (Figure S7D–F). To confirm the involvement of NRF-2, we transfected LLC or H1650 with siRNA for NRF-2 or scramble and then treated these cells with WFA. We used western blotting to confirm that NRF-2 siRNA indeed inhibits the activation of NRF-2 (Figure S7I). Unlike our previous findings, we found that WFA still upregulated PD-L1 in NRF-2 knockdown cells similar to the scramble controls (Figure S7G,H). Due to the discrepancy in our findings between brusatol and NRF-2 KO and the lack of specificity of the pharmacologic inhibitors, we conclude that NRF-2 may not be essential in WFA-induced PD-L1 upregulation. Further studies are needed to elucidate the downstream regulators of WFA mediated PD-L1 upregulation. In summary, we conclude that WFA mediates PD-L1 and ecto-CRT expression mainly by inducing ROS production in vitro.

### 3.5. WFA Sensitizes LLC Syngeneic Mouse Tumors to α-PD-L1 In Vivo

**Figure 5:**
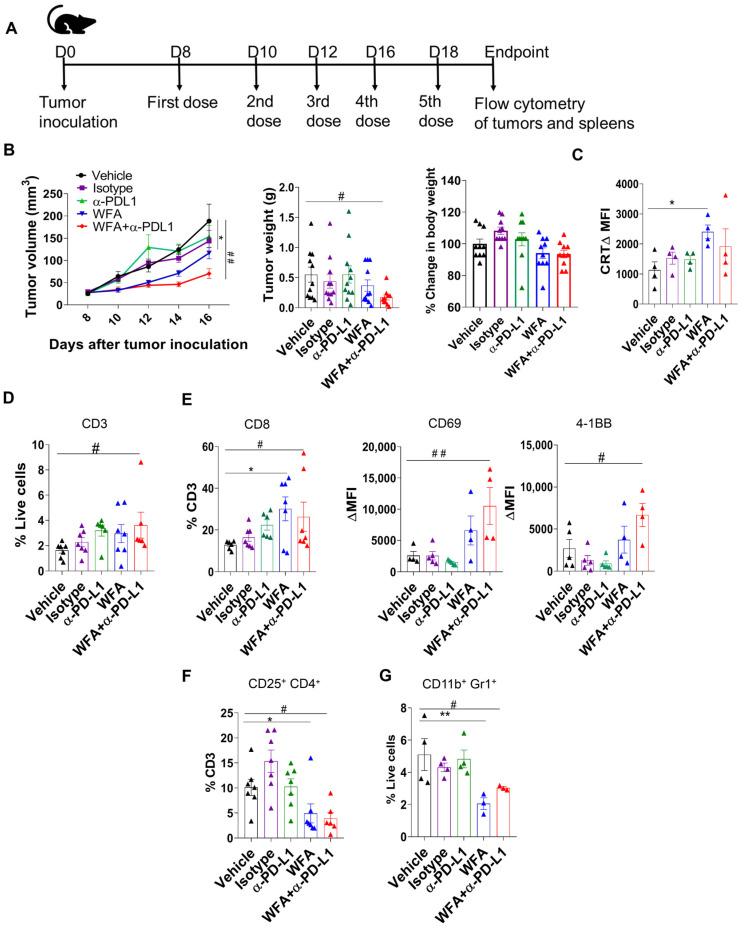
WFA sensitizes flank tumors to α-PD-L1 therapy and targets immunosuppressive MDSCs and T-regs. (**A**) Establishment of LLC flank tumors and treatment schedule. (**B**) Tumor volume measurement in mm^3^ tumor weight, and percent change in body weight compared to the vehicle control at the endpoint of the experiment. (**C**) Change in the levels of ecto-CRT in tumors was measured using flow cytometry. (**D**–**G**) Flow cytometry was used to determine the change in immune cell infiltration in collected tumors. WFA treatment increased CD3 (**D**) and CD8 T-cell (**E**) infiltration and activation markers 4-1BB and CD69. Moreover, WFA reduced the immunosuppressive T regs (**F**) and CD11b^+^ Gr1^+^ MDSC (**G**) populations. The means of two independent experiments ± SEM are shown, and the statistical significance was calculated using one-way ANOVA and a Fisher LSD post hoc test. * and # represent *p* < 0.05 and ** and ## represent *p* < 0.01. * Symbols refer to the difference between vehicle control and WFA, while # refers to comparisons between the vehicle control and WFA+α-PD-L1 combination.

Since our in vitro data showed that WFA could sensitize NSCLC cells to α-PD-L1, we tested the effectiveness of a WFA and α-PD-L1 combination therapy in a LLC syngeneic mouse model. C57BL/6 mice were injected with 5 × 10^5^ LLC cells per flank and the treatment started eight days after tumor initiation (when the tumors were first palpable). Mice were randomized into vehicle control (10% DMSO, 90% Glyceryl Tri octanoate), isotype control (200 µg/mouse dose), WFA (4 mg/kg), α-PD-L1 (200 µg/mouse dose), or WFA+α-PD-L1 combination and treated every other day with a total of five treatments (Figure 5A). While α-PD-L1 did not change the tumor size, WFA showed a non-significant reduction in tumor size (Figure 5B). Although the individual treatments did not change tumor size, the combination treatment significantly reduced tumor growth (Figure 5B). Moreover, we measured the body weight of the mice as an indicator of toxicity/disease burden. Interestingly, we found that the combination treatment did not significantly alter the body weight from the control mice (Figure 5B and Figure S6C). At the endpoint, mice were euthanized for spleen and tumor collection and dissociation. To investigate the mechanism by which WFA increased the effectiveness of α-PD-L1 in vivo, we analyzed the changes in tumor-immune infiltration induced by different treatments, including both effector cell and immunosuppressive cell populations (Figure S6A,B). Similar to our in vitro findings, WFA increased the levels of ecto-CRT in vivo compared to the control mice (Figure 5C). Moreover, Flow cytometry analysis shows that WFA treatment skewed the TME towards a more anti-tumor phenotype by increasing CD8 T-cell infiltration (Figure 5D,E). However, only the combination treatment was observed to significantly increase the expression of T-cell activation markers CD69 and 41BB compared to vehicle control (Figure 5E). Additionally, WFA targets immunosuppressive cell populations including CD11b+Gr1+MDSCs and CD25+CD4+T-regs, reducing their presence in the tumor (Figure 5F,G). In summary, our in vivo results show that WFA sensitizes LLC tumors to α-PD-L1 and elicits an anti-tumor immune response by increasing CTL infiltration and targeting immunosuppressive cells.

### 3.6. ROS Plays a Role in Inducing the Effectiveness of WFA+α-PD-L1 Combination

**Figure 6:**
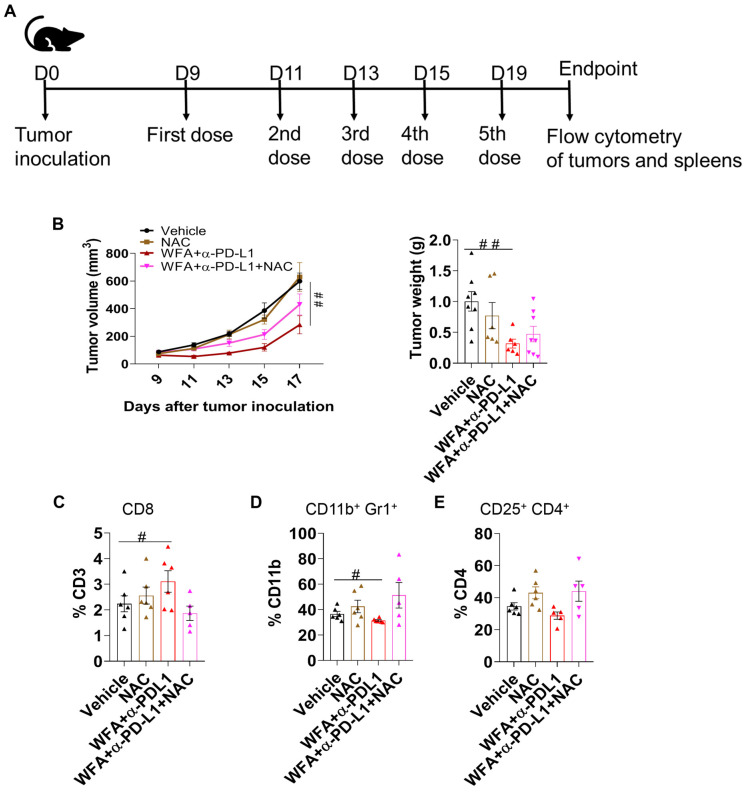
ROS partially contributes to WFA-mediated anti-cancer effectiveness and immunomodulation in vivo. (**A**) Establishment of LLC flank tumors and treatment schedule. (**B**) Tumor volume measurement in mm^3^ and tumor weight at the endpoint of the experiment. (**C**–**E**) Flow cytometry was used to determine the change in immune cell infiltration in collected tumors. NAC treatment reversed WFA mediated increase in CD8 T-cell (**C**) infiltration while increasing the immunosuppressive CD11b+Gr1+MDSCs (**D**) and CD25+CD4+T-reg (**E**) populations. Statistical significance was calculated using one-way ANOVA and each group was compared to the vehicle control using the Fisher LSD post hoc test where # represents *p* < 0.05 and ## represents a *p* < 0.01.

Since ROS production was responsible for inducing PD-L1 expression in vitro, we wanted to confirm whether WFA-mediated ROS production plays a role in tumor immunomodulation in vivo. To confirm the role of ROS, we treated LLC syngeneic mice with WFA+α-PD-L1 or WFA+α-PD-L1+NAC (100 mg/kg) (Figure 6A). Similar to our previous findings, we found that the WFA+anti-PD-L1 combination significantly reduced tumor growth (Figure 6B). However, the mice that received the added NAC treatment showed reduced effectiveness of the combination treatment. However, the change was not statistically significant from the WFA+α-PD-L1 combination treatment. At the endpoint, tumors and spleens were collected to measure the effect of NAC on tumor immune infiltration. Interestingly, we found that NAC treatment reduced CD8 T-cell infiltration (Figure 6C) and increased the immunosuppressive cell populations when added to the WFA+α-PD-L1 combination (Figure 6D,E). In summary, although NAC did not completely reverse the effectiveness of the WFA+α-PD-L1 treatment, its partial rescue of the phenotype demonstrates that ROS plays a role in WFA mediated anti-tumor immunomodulation in vivo.

## 4. Discussion

A major finding of our study is that WFA induces ICD, increases the release of DAMPs, and increases PD-L1 expression in NSCLC, in addition to colorectal and breast cancer cells. We found that WFA-induced ICD in both murine and human NSCLC cell lines was characterized by the increased surface expression of CRT and the release of HMGB-1. These molecules act as a ‘find me’ signal, attracting APC to the tumor site, and an ‘eat me’ signal that promotes DC activation and engulfment of tumor antigens [41]. Consequently, activated APC can present tumor antigens to prime an anti-tumor T-cell response [43,44]. We confirmed that WFA-induced DAMP release from treated LLC murine cell line leads to the increased activation of APC represented by syngeneic bone marrow-derived DC.

Another major finding of our study is the role of ROS in inducing PD-L1 expression in response to WFA in different types of cancer cell lines. Other studies showed that WFA causes cancer cell apoptosis by inducing mitochondrial dysfunction by inhibiting complex III of the electron transport chain, which in turn increases the intracellular levels of ROS [31]. Interestingly, this increase is not observed in non-cancerous cells, providing a potential explanation of WFA’s selective toxicity [45,46]. We tested whether ROS plays a role in WFA-mediated PD-L1 increase by treating the cells with WFA and a ROS scavenger (NAC or GSH). Notably, we found that either NAC or GSH was able to completely reverse PD-L1 upregulation. Additionally, ROS scavengers inhibited WFA-induced increase in ecto-CRT. These findings prove that ROS plays a key role in WFA-mediated immunomodulation including ICD and PD-L1 upregulation. The literature shows that ROS can play either an immunostimulatory or an immunoinhibitory effect in the TME based on the type of cells from which it is produced and their specific location within the TME [47,48]. While ROS can induce ICD, increase tumor antigenicity, and reprogram tumor-associated macrophages, it also represents a major immunosuppressive mechanism when released by MDSCs or T-regs [49,50,51,52,53]. However, WFA was found to inhibit MDSCs production of ROS in a 4T1 mouse model suggesting that WFA plays a dual role in ROS signaling based on the cellular context [36].

Although the relationship between ROS and PD-L1 is complex, a few studies show that ROS usually induces PD-L1 upregulation by activating certain downstream transcription factors such as YAP-1, HIF-1α, and NF-κB [51,54,55]. However, there are ROS-inducing drugs that reduce PD-L1 expression and vice versa [56,57]. We attempted to investigate the downstream regulator responsible for ROS-mediated PD-L1 upregulation using the IPA. By applying the mRNA expression fold change values from a publicly available dataset of WFA-treated MCF-7 breast cancer cells to a signaling network including ROS-PD-L1 connections, we found that both ROS and PD-L1 are predicted to be increased by WFA treatment. Additionally, IPA predictions show that multiple downstream regulators might be involved in ROS-mediated PD-L1 upregulation including STAT-3, YAP-1, and HSF-1 (Figure S5). While testing all potential pathways is beyond the scope of this study, we pragmatically selected a couple to examine the ROS-mediated-PD-L1 upregulation. First, the combination of WFA with the pharmacologic inhibitors of STAT-3 or HIF1α did not alter PD-L1 upregulation, suggesting that the pathways involving these transcription factors are not involved. In contrast, the NRF-2 inhibitor brusatol reversed PD-L1 upregulation, suggesting its role in WFA-induced PD-L1 upregulation, which is consistent with another study [58]. However, siRNA for NRF-2 did not rescue PD-L1 upregulation, questioning the role of NRF-2 in this process. Given the prediction of multiplicity of pathways involved in ROS-mediated PD-L1 upregulation, we infer that WFA-induced PD-L1 upregulation may involve more than a single pathway.

As our in vitro results show that WFA promotes ICD and PD-L1 upregulation, we tested the effectiveness of WFA+α-PD-L1 combination therapy in an in vivo LLC immunocompetent model. As others have shown, α-PD-L1 did not reduce tumor growth [59,60]. Although WFA-treated mice showed a reduction in tumor growth, the change was non-significant. The combination treatment of WFA+α-PD-L1 significantly reduced tumor growth. Moreover, flow cytometry analysis of tumor immune infiltration shows that WFA increased T-cell infiltration and reduced the immunosuppressive cell populations. We found that WFA increased CD8 T-cell infiltration and appeared to increase activation, however, the change in T-cell activation was not significant. The combination treatment showed a significant increase in CD8 T-cell percentages and activation markers CD69 and 4-1BB. We also found that both WFA and combination treatments reduced the levels of immunosuppressive MDSCs and T-regs. Overall, WFA can change a cold TME into a hot TME, increasing the effectiveness of α-PD-L1 in NSCLC [17,61]. Due to the opposing effects of ROS on anti-tumor immunity, we further investigated the role of ROS in WFA-mediated immunomodulation in vivo. Treating the LLC mice with NAC partially reversed the tumor growth inhibition observed with the WFA+α-PD-L1 combination treatment. Our flow cytometry analysis shows that NAC also reversed the immune phenotype observed with the combination treatment by reducing CD8 T-cell and increasing MDSC and T-reg infiltration. However, the change of tumor growth was not statistically significant from the combination treatment, which indicates that ROS only plays a partial role in WFA in vivo effectiveness. That suggests the involvement of other pathways or factors in the TME that may not be connected to ROS in WFA-mediated anti-cancer effectiveness. Further studies are required to identify these pathways. Although we observed WFA-mediated immunomodulation was ubiquitous in different types of cancer cells, further in vivo studies are needed to confirm the effectiveness of WFA and α-PD-L1 combination therapy in colorectal and breast cancer. Additionally, further studies are needed to investigate the effect of WFA on additional IC molecules to identify other effective combinatorial approaches. The choice of proper combinatorial therapy allows for personalized therapy. We monitored body weights as an indicator of toxicity and found that the WFA+α-PD-L1 combination treatment did not change the body weight compared to the vehicle treated controls. This suggests that the combination of WFA+α-PD-L1 may be safe and tolerable, however, this needs to be tested clinically.

## 5. Conclusions

To summarize, our results demonstrate that WFA induces ICD in NSCLC and colorectal cancer cell lines and increases the release of DAMPs. Moreover, we found that WFA treatment increases PD-L1 expression in NSCLC, colorectal, and breast cancer cell lines. We investigated the underlying mechanism and found that these changes were mediated by ROS and were reversed by ROS scavengers like NAC and GSH. Moreover, for the first time, we showed that WFA sensitizes NSCLC to α-PD-L1 in an in vivo mouse model without causing additional toxicity. Our results provide a new combinatorial approach that can improve patient response to ICBs by converting an immunologically cold to an immunologically hot TME and prompts testing its effectiveness in a clinical setting.

## Data Availability

The dataset we analyzed to construct the molecular connection involved in ROS-mediated PD-L1 upregulation by WFA is available on the Gene Omnibus Database and can be accessed using the GEO accession GSE53049.

## Supplementary Materials

The following supporting information can be downloaded at: https://www.mdpi.com/article/10.3390/cancers15123089/s1, File S1: The original western blot figures; Table S1: List of antibodies and reagents used in flow cytometry experiments; Table S2: List of primer sequences used in qRT-PCR; Table S3: List of antibodies used in western blotting; Figure S1: qRT-PCR analysis of the change in expression of pro-apoptotic and anti-apoptotic induced by WFA treatment; Figure S2: WFA induced ICD and PD-L1 upregulation in colorectal cancer cell lines; Figure S3: The gating strategy used for the DC ex-vivo activation assay. Hierarchical gating was used to identify the total cell population followed by single live cells the CD11C+ DC. Out of the CD11C population, the expression of DC cell activation markers was determined based on the fluorescence minus one (FMO) controls; Figure S4: WFA treatment increased the mRNA levels of IFNα; Figure S5: A gene interaction network between the PDL1 signaling pathway and reactive oxygen species (nitric oxide, oxygen radical, hydrogen peroxide, and lipoxygenase) signaling was generated using known connections obtained from the Ingenuity Pathway Analysis database; Figure S6: The gating strategy used to determine tumor immune infiltration in LLC tumors and spleens; Figure S7: Investigating the downstream molecular regulators that may be involved in WFA-mediated PD-L1 upregulation.

## References

[1] Siegel R.L., Miller K.D., Fuchs H.E., Jemal A. (2022). Cancer statistics, 2022. CA A Cancer J. Clin..

[2] Centers for Disease Control and Prevention (2022). U.S. Cancer Statistics Lung Cancer Stat Bite.

[3] He X., Xu C. (2020). Immune checkpoint signaling and cancer immunotherapy. Cell Res..

[4] Sánchez-Paulete A.R., Teijeira A., Cueto F.J., Garasa S., Pérez-Gracia J.L., Sánchez-Arráez A., Sancho D., Melero I. (2017). Antigen cross-presentation and T-cell cross-priming in cancer immunology and immunotherapy. Ann. Oncol..

[5] Chambers C.A., Allison J.P. (1999). Costimulatory regulation of T cell function. Curr. Opin. Cell Biol..

[6] Borghaei H., Paz-Ares L., Horn L., Spigel D.R., Steins M., Ready N.E., Chow L.Q., Vokes E.E., Felip E., Holgado E. (2015). Nivolumab versus Docetaxel in Advanced Nonsquamous Non–Small-Cell Lung Cancer. N. Engl. J. Med..

[7] Principe N., Kidman J., Goh S., Tilsed C.M., Fisher S.A., Fear V.S., Forbes C.A., Zemek R.M., Chopra A., Watson M. (2020). Tumor Infiltrating Effector Memory Antigen-Specific CD8(+) T Cells Predict Response to Immune Checkpoint Therapy. Front. Immunol..

[8] Tang S., Qin C., Hu H., Liu T., He Y., Guo H., Yan H., Zhang J., Tang S., Zhou H. (2022). Immune Checkpoint Inhibitors in Non-Small Cell Lung Cancer: Progress, Challenges, and Prospects. Cells.

[9] Wellenstein M.D., de Visser K.E. (2018). Cancer-Cell-Intrinsic Mechanisms Shaping the Tumor Immune Landscape. Immunity.

[10] Bai R., Chen N., Li L., Du N., Bai L., Lv Z., Tian H., Cui J. (2020). Mechanisms of Cancer Resistance to Immunotherapy. Front. Oncol..

[11] Bai R., Lv Z., Xu D., Cui J. (2020). Predictive biomarkers for cancer immunotherapy with immune checkpoint inhibitors. Biomark. Res..

[12] Kowanetz M., Zou W., Gettinger Scott N., Koeppen H., Kockx M., Schmid P., Kadel Edward E., Wistuba I., Chaft J., Rizvi Naiyer A. (2018). Differential regulation of PD-L1 expression by immune and tumor cells in NSCLC and the response to treatment with atezolizumab (anti–PD-L1). Proc. Natl. Acad. Sci. USA.

[13] Wang L., Hu Y., Wang S., Shen J., Wang X. (2020). Biomarkers of immunotherapy in non-small cell lung cancer (Review). Oncol. Lett..

[14] Sahin I.H., Akce M., Alese O., Shaib W., Lesinski G.B., El-Rayes B., Wu C. (2019). Immune checkpoint inhibitors for the treatment of MSI-H/MMR-D colorectal cancer and a perspective on resistance mechanisms. Br. J. Cancer.

[15] Spaas M., Lievens Y. (2019). Is the Combination of Immunotherapy and Radiotherapy in Non-small Cell Lung Cancer a Feasible and Effective Approach?. Front. Med..

[16] Shao X., Wang X., Guo X., Jiang K., Ye T., Chen J., Fang J., Gu L., Wang S., Zhang G. (2019). STAT3 Contributes To Oncolytic Newcastle Disease Virus-Induced Immunogenic Cell Death in Melanoma Cells. Front. Oncol..

[17] Liu Y.T., Sun Z.J. (2021). Turning cold tumors into hot tumors by improving T-cell infiltration. Theranostics.

[18] Luo M., Wang F., Zhang H., To K.K.W., Wu S., Chen Z., Liang S., Fu L. (2020). Mitomycin C enhanced the efficacy of PD-L1 blockade in non-small cell lung cancer. Signal Transduct. Target. Ther..

[19] Fournel L., Wu Z., Stadler N., Damotte D., Lococo F., Boulle G., Ségal-Bendirdjian E., Bobbio A., Icard P., Trédaniel J. (2019). Cisplatin increases PD-L1 expression and optimizes immune check-point blockade in non-small cell lung cancer. Cancer Lett..

[20] Florescu M., Cinteza M., Vinereanu D. (2013). Chemotherapy-induced Cardiotoxicity. Maedica.

[21] Li C., Bhatti S.A., Ying J. (2022). Immune Checkpoint Inhibitors-Associated Cardiotoxicity. Cancers.

[22] Dutta R., Khalil R., Green R., Mohapatra S.S., Mohapatra S. (2019). Withania Somnifera (Ashwagandha) and Withaferin A: Potential in Integrative Oncology. Int. J. Mol. Sci..

[23] Kakar S.S., Parte S., Carter K., Joshua I.G., Worth C., Rameshwar P., Ratajczak M.Z. (2017). Withaferin A (WFA) inhibits tumor growth and metastasis by targeting ovarian cancer stem cells. Oncotarget.

[24] Kyakulaga A.H., Aqil F., Munagala R., Gupta R.C. (2018). Withaferin A inhibits Epithelial to Mesenchymal Transition in Non-Small Cell Lung Cancer Cells. Sci. Rep..

[25] Ting L.-L., Chou A.S.-B., Hsieh C.-H., Hsiung S.-C., Pang S.-T., Liao S.-K. (2016). Withaferin A targeting both cancer stem cells and metastatic cancer stem cells in the UP-LN1 carcinoma cell model. J. Cancer Metastasis Treat..

[26] Shiragannavar V.D., Gowda N.G.S., Kumar D.P., Mirshahi F., Santhekadur P.K. (2020). Withaferin A Acts as a Novel Regulator of Liver X Receptor-α in HCC. Front. Oncol..

[27] Heyninck K., Lahtela-Kakkonen M., Van der Veken P., Haegeman G., Vanden Berghe W. (2014). Withaferin A inhibits NF-kappaB activation by targeting cysteine 179 in IKKβ. Biochem. Pharm..

[28] Lee I.C., Choi B.Y. (2016). Withaferin-A—A Natural Anticancer Agent with Pleitropic Mechanisms of Action. Int. J. Mol. Sci..

[29] Kunimasa K., Nagano T., Shimono Y., Dokuni R., Kiriu T., Tokunaga S., Tamura D., Yamamoto M., Tachihara M., Kobayashi K. (2017). Glucose metabolism-targeted therapy and withaferin A are effective for epidermal growth factor receptor tyrosine kinase inhibitor-induced drug-tolerant persisters. Cancer Sci..

[30] Ghosh K., De S., Das S., Mukherjee S., Sengupta Bandyopadhyay S. (2016). Withaferin A Induces ROS-Mediated Paraptosis in Human Breast Cancer Cell-Lines MCF-7 and MDA-MB-231. PLoS ONE.

[31] Sehrawat A., Samanta S.K., Hahm E.R., St Croix C., Watkins S., Singh S.V. (2019). Withaferin A-mediated apoptosis in breast cancer cells is associated with alterations in mitochondrial dynamics. Mitochondrion.

[32] Guo R., Gan L., Lau W.B., Yan Z., Xie D., Gao E., Christopher T.A., Lopez B.L., Ma X., Wang Y. (2019). Withaferin A Prevents Myocardial Ischemia/Reperfusion Injury by Upregulating AMP-Activated Protein Kinase-Dependent B-Cell Lymphoma2 Signaling. Circ. J..

[33] Liu X., Chen L., Liang T., Tian X.D., Liu Y., Zhang T. (2017). Withaferin A induces mitochondrial-dependent apoptosis in non-small cell lung cancer cells via generation of reactive oxygen species. J. BUON.

[34] Malik F., Singh J., Khajuria A., Suri K.A., Satti N.K., Singh S., Kaul M.K., Kumar A., Bhatia A., Qazi G.N. (2007). A standardized root extract of *Withania somnifera* and its major constituent withanolide-A elicit humoral and cell-mediated immune responses by up regulation of Th1-dominant polarization in BALB/c mice. Life Sci..

[35] Tharakan A., Shukla H., Benny I.R., Tharakan M., George L., Koshy S. (2021). Immunomodulatory Effect of *Withania somnifera* (Ashwagandha) Extract-A Randomized, Double-Blind, Placebo Controlled Trial with an Open Label Extension on Healthy Participants. J. Clin. Med..

[36] Sinha P., Ostrand-Rosenberg S. (2013). Myeloid-derived suppressor cell function is reduced by Withaferin A, a potent and abundant component of Withania somnifera root extract. Cancer Immunol. Immunother..

[37] Hossain D.M.S., Javaid S., Cai M., Zhang C., Sawant A., Hinton M., Sathe M., Grein J., Blumenschein W., Pinheiro E.M. (2018). Dinaciclib induces immunogenic cell death and enhances anti-PD1-mediated tumor suppression. J. Clin. Investig..

[38] Kepp O., Menger L., Vacchelli E., Locher C., Adjemian S., Yamazaki T., Martins I., Sukkurwala A.Q., Michaud M., Senovilla L. (2013). Crosstalk between ER stress and immunogenic cell death. Cytokine Growth Factor Rev..

[39] Fucikova J., Kepp O., Kasikova L., Petroni G., Yamazaki T., Liu P., Zhao L., Spisek R., Kroemer G., Galluzzi L. (2020). Detection of immunogenic cell death and its relevance for cancer therapy. Cell Death Dis..

[40] Nath S., Obaid G., Hasan T. (2019). The Course of Immune Stimulation by Photodynamic Therapy: Bridging Fundamentals of Photochemically Induced Immunogenic Cell Death to the Enrichment of T-Cell Repertoire. Photochem. Photobiol..

[41] Galluzzi L., Kepp O., Hett E., Kroemer G., Marincola F.M. (2023). Immunogenic cell death in cancer: Concept and therapeutic implications. J. Transl. Med..

[42] Xia S., Miao Y., Liu S. (2018). Withaferin A induces apoptosis by ROS-dependent mitochondrial dysfunction in human colorectal cancer cells. Biochem. Biophys. Res. Commun..

[43] Krysko D.V., Garg A.D., Kaczmarek A., Krysko O., Agostinis P., Vandenabeele P. (2012). Immunogenic cell death and DAMPs in cancer therapy. Nat. Rev. Cancer.

[44] Di Blasio S., Wortel I.M., van Bladel D.A., de Vries L.E., Duiveman-de Boer T., Worah K., de Haas N., Buschow S.I., de Vries I.J., Figdor C.G. (2016). Human CD1c^+^ DCs are critical cellular mediators of immune responses induced by immunogenic cell death. Oncoimmunology.

[45] Batumalaie K., Amin M.A., Murugan D.D., Sattar M.Z.A., Abdullah N.A. (2016). Withaferin A protects against palmitic acid-induced endothelial insulin resistance and dysfunction through suppression of oxidative stress and inflammation. Sci. Rep..

[46] Zhou Z., Xiang W., Jiang Y., Tian N., Wei Z., Wen X., Wang W., Liao W., Xia X., Li Q. (2020). Withaferin A alleviates traumatic brain injury induced secondary brain injury via suppressing apoptosis in endothelia cells and modulating activation in the microglia. Eur. J. Pharmacol..

[47] Bailly C. (2020). Regulation of PD-L1 expression on cancer cells with ROS-modulating drugs. Life Sci..

[48] Choi E.J., Jeon C.H., Lee I.-K. (2022). Ferric Ammonium Citrate Upregulates PD-L1 Expression through Generation of Reactive Oxygen Species. J. Immunol. Res..

[49] Roux C., Jafari S.M., Shinde R., Duncan G., Cescon D.W., Silvester J., Chu M.F., Hodgson K., Berger T., Wakeham A. (2019). Reactive oxygen species modulate macrophage immunosuppressive phenotype through the up-regulation of PD-L1. Proc. Natl. Acad. Sci. USA.

[50] Xue J., Li L., Li N., Li F., Qin X., Li T., Liu M. (2019). Metformin suppresses cancer cell growth in endometrial carcinoma by inhibiting PD-L1. Eur. J. Pharmacol..

[51] Zhou X., Wang W., Wang C., Zheng C., Xu X., Ni X., Hu S., Cai B., Sun L., Shi K. (2019). DPP4 Inhibitor Attenuates Severe Acute Pancreatitis-Associated Intestinal Inflammation via Nrf2 Signaling. Oxidative Med. Cell. Longev..

[52] Zhu S., Liu S., Wang L., Ding W., Sha J., Qian H., Lu Y. (2019). Brusatol Protects HepG2 Cells against Oxygen-Glucose Deprivation-Induced Injury via Inhibiting Mitochondrial Reactive Oxygen Species-Induced Oxidative Stress. Pharmacology.

[53] Kotsafti A., MScarpa, Castagliuolo I., Scarpa M. (2020). Reactive Oxygen Species and Antitumor Immunity-From Surveillance to Evasion. Cancers.

[54] Wang L., Kuang Z., Zhang D., Gao Y., Ying M., Wang T. (2021). Reactive oxygen species in immune cells: A new antitumor target. Biomed. Pharmacother..

[55] Hassannia B., Wiernicki B., Ingold I., Qu F., Van Herck S., Tyurina Y.Y., Bayır H., Abhari B.A., Angeli J.P.F., Choi S.M. (2018). Nano-targeted induction of dual ferroptotic mechanisms eradicates high-risk neuroblastoma. J. Clin. Investig..

[56] Ohl K., Tenbrock K. (2018). Reactive Oxygen Species as Regulators of MDSC-Mediated Immune Suppression. Front. Immunol..

[57] Shi C., Liu T., Guo Z., Zhuang R., Zhang X., Chen X. (2018). Reprogramming Tumor-Associated Macrophages by Nanoparticle-Based Reactive Oxygen Species Photogeneration. Nano Lett..

[58] Zhu B., Tang L., Chen S., Yin C., Peng S., Li X., Liu T., Liu W., Han C., Stawski L. (2018). Targeting the upstream transcriptional activator of PD-L1 as an alternative strategy in melanoma therapy. Oncogene.

[59] Luo F., Luo M., Rong Q.-X., Zhang H., Chen Z., Wang F., Zhao H.-Y., Fu L.-W. (2019). Niclosamide, an antihelmintic drug, enhances efficacy of PD-1/PD-L1 immune checkpoint blockade in non-small cell lung cancer. J. Immunother. Cancer.

[60] Veronica Olivo P., Damiënne M., Alexander M.A.v.d.W., Natasja G.L., Rianne B., Relinde I.Y.L., Dario N., Jan T., Ala Y., Ludwig J.D. (2021). Releasing the brakes of tumor immunity with anti-PD-L1 and pushing its accelerator with L19–IL2 cures poorly immunogenic tumors when combined with radiotherapy. J. Immunother. Cancer.

[61] Duan Q., Zhang H., Zheng J., Zhang L. (2020). Turning Cold into Hot: Firing up the Tumor Microenvironment. Trends Cancer.

