# Supplementary material for: Withaferin A Increases the Effectiveness of Immune Checkpoint Blocker for the Treatment of Non-Small Cell Lung Cancer
Source: Cancers (Basel). 2023 Jun 7;15(12):3089. doi: 10.3390/cancers15123089 (PMC10295988; doi:10.3390/cancers15123089)
Supplement: Supplementary file 1 [file cancers-15-03089-s001.zip › supplementary figures and tables.pdf]

Withaferin A Increases the Effectiveness of Immune Checkpoint Blocker for the Treatment of Non-Small Cell Lung Cancer

Supplemental Data

Supplemental Table S1. List of antibodies and reagents used in flow cytometry experiments.

Antibody	Catalog #	Clone	Antibody	Catalog #	Clone
Alexa Fluor® 488 anti-mouse CD45	103121	30-F11	PE/Dazzle™ 594 anti-mouse Ly-6G	127647	1A8
Alexa Fluor® 647 anti-mouse F4/80	123121	BM8	PE/Cy5 anti-mouse I-A/I-E	107611	M5/114.15.2
APC/Fire™ 750 anti-mouse/human CD11b	101261	M1/70	PE anti-mouse CD86	159203	A17199A
BV421 cd80	104725	16-10A1	Alexa Fluor® 488 anti-mouse NK-1.1	108717	PK136
Brilliant Violet 570™ anti-mouse CD11c	117331	N418	Alexa Fluor® 700 anti-mouse CD3	100215	17A2
Brilliant Violet 650™ anti-mouse CD206 (MMR)	141723	C068C2	APC anti-mouse CD279 (PD-1)	135209	29F.1A12
PE/Cy7 anti-mouse CD103	121425	2E7	Brilliant Violet 570™ anti-mouse CD4	100541	RM4-5
Brilliant Violet 605™ anti-mouse CD8a	100743	53-6.7	Anti-Calreticulin antibody	Ab2907	Polyclonal
Brilliant Violet 650™ anti-mouse CD69	104541	H1.2F3	PE/Cy5 anti-mouse CD25	102010	PC61
PE anti-mouse CD137 (4-1BB)	106105		Alexa Fluor® 700 anti-mouse Ly-6C	128023	HK1.4
IgG (H+L) Cross-Adsorbed Secondary Antibody, Alexa Fluor™ 488	A11008	Polyclonal	PE anti-mouse CD274 (B7-H1, PD-L1) Antibody	124307	10F.9G2
PE Rat IgG2b, κ Isotype Ctrl Antibody	400607	RTK4530	PE anti-human CD274 (B7-H1, PD-L1) Antibody	329705	29E.2A3
PE Mouse IgG2b, κ Isotype Ctrl Antibody	400311	MPC-11	Zombie Aqua™ Fixable Viability Kit	423101	
OneComp eBeads™ Compensation Beads	01-1111-41				

Supplemental Table S2. List of primer sequences used in qRT-PCR.

Gene name	Species	Sequence
Pd-l1	Mouse	5′- GTG AAA CCC TGA GTC TTA TCC -3′<br>5′- GAC CAT TCT GAG ACA ATT CC -3′
PD-L1	Human	5′- TGC CGA CTA CAA GCG AAT TAC TG -3′<br>5′- CTG CTT GTC CAG ATG ACT TCG G -3′
Bax	Mouse	5′- GCG TGG TTG CCC TCT TCT ACT TTG -3′<br>5′- AGT CCA GTG TCC AGC CCA TGA TG -3′
BAX	Human	5′- TCC AGG ATC GAC CAG GGC GA -3′<br>5′- AAA AGG GCG ACA ACC CGG CC -3′
Bcl-2	Mouse	5′- GTC CCG CCT CTT CAC TCA G -3′<br>5′- GAT TCT GGT GTT TCC CCG TTG G -3′
BCL-2	Human	5′- CTC CCT CTC CCC GCC ACT CC -3′<br>5′- GGG GGT GTC TTC AAT CAC GCG -3′
Bak-1	Mouse	5′- AAG GTG GGC TGC GAT GAG TCC -3′<br>5′- GGG TCT CCT GCT GGT C -3′
BAD	Human	5′- GGG ATG GGG GAG GAG CCC AG -3′<br>5′- AAG GTC ACT GGG AGG GGG CG -3′
IFNα	Mouse	5′- GGA CTT TGG ATT CCC GCA GGA GAA G -3′<br>5′- GCT GCA TCA GAC AGC CTT GCA GGT C -3′
IFNα	Human	5′- GAC TTC ATC TTG GCT GTG A -3′<br>5′- TGA TTT CTG CTC TGA CAA CCT -3′
GAPDH	Mouse	5′- CCT GGT ATG ACA ATG AAT ACG GC -3′<br>5′- CTC CTT GGA GGC CAT GTA GG -3′
GAPDH	Human	5′- CGA CAG TCA GCC GCA TCT T -3′<br>5′- CCC CAT GGT GTC TTC AAT CAC GCG -3′
B-actin	Mouse	5′- AAA TCT GGC ACC ACA CCT TC -3′<br>5′- GGG GTG TTG AAG GTC TCA AA -3′
B-ACTIN	Human	5′- TTC TAC AAT GAC CTG GGT GTG -3′<br>5′- GGG GTG TTG AAG GTC TCA AA -3′

Supplemental Table S3. List of antibodies used in western blotting.

Antibody	Catalog number	Antibody	Catalog number
beta Actin antibody	SC-47778	PD-L1/CD274 Rabbit pAb	A1645
Vinculin antibody	SC-25336	PD-L1 (E1L3N®) XP® Rabbit mAb	13684
mouse anti-rabbit IgG-HRP	SC-2357	CHOP (L63F7) Mouse mAb	2895
m-IgGκ BP-HRP	SC-516102	eIF2α (D7D3) XP® Rabbit mAb	5324
Phospho-eIF2α (Ser51) (D9G8) XP® Rabbit mAb	3398	Nrf2 Polyclonal Antibody	PA5-88084

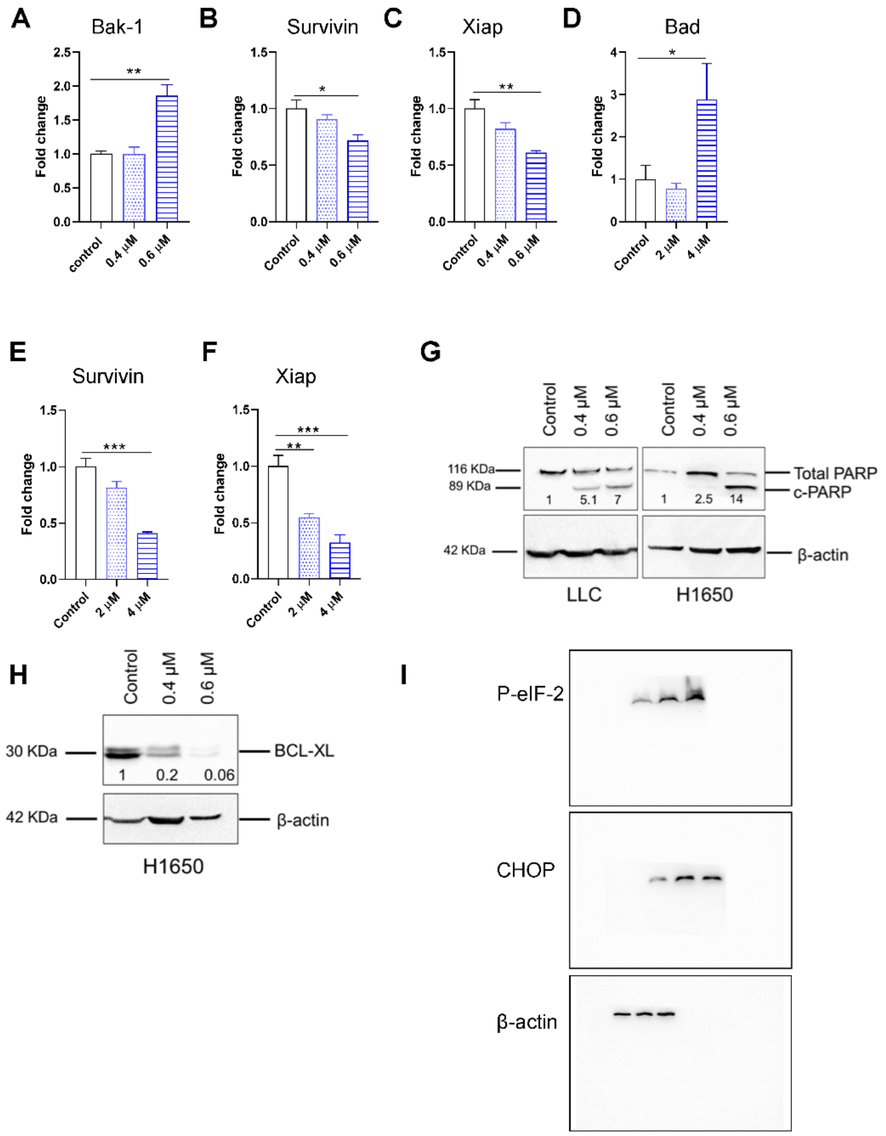

Figure S1. qRT-PCR analysis of the change in expression of pro-apoptotic and anti-apoptotic induced by WFA treatment. WFA increased the mRNA levels of proapoptotic protein Bak-1 in (**A**) LLC cells and BAD in (**C**) H1650 cells. WFA also decreased the mRNA levels of the anti-apoptotic protein Survivin and Xiap in both (**B**) LLC and (**D**) A549cell line. PCR was repeated twice, and one representative experiment is shown as mean ± SEM of 3 technical replicates. Western blotting shows that WFA treatment increased PARP cleavage in (**E**) LLC and H1650 cells and decreases the anti-apoptotic protein BCL-XL in (**F**) H1650 cells. (**G–I**) Western blots. One-way ANOVA and Fisher LSD post hoc test were used to compare the treatment groups to the control. * $p < 0.05$, ** $p < 0.01$, *** $p < 0.001$.

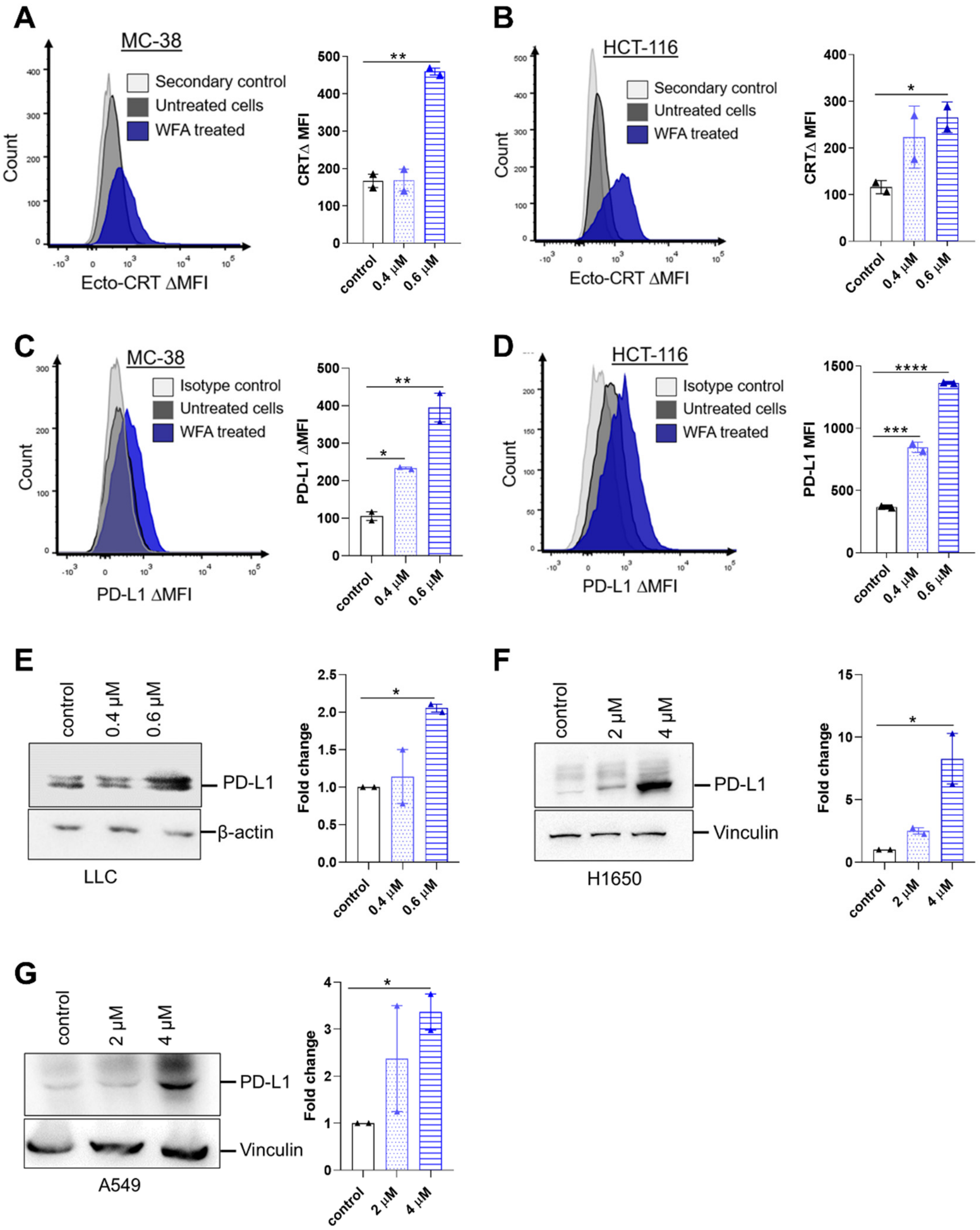

Figure S2. WFA induced ICD and PD-L1 upregulation in colorectal cancer cell lines. WFA increased the levels of ecto-CRT in the murine colorectal cancer cell line (**A**) MC-38 and human cell line (**B**) HCT-116. WFA increased the expression of PD-L1 in both (**C**) MC-38 and (**D**) HCT-116 cells. (**E**) Western blotting shows that WFA treatment increases the levels of PD-L1 protein

in LLC, H1650 and A549 NSCLC cell lines. H1650 cells (at a higher density) were treated with a higher concentration of WFA (2 and 4 μM) to facilitate cell collection and protein isolation. The band density was quantified using Image J software and normalized to the housekeeping protein. The fold change from untreated control was averaged from two independent experiments ± SEM and one-way ANOVA was used to calculate the statistical significance. A Fisher LSD post hoc test was performed to compare the treatments to the control in (**E**) LLC, (**F**) H1650, (**G**) A549 cells. * $p < 0.05$, ** $p < 0.01$, *** $p < 0.001$, **** $p < 0.0001$.

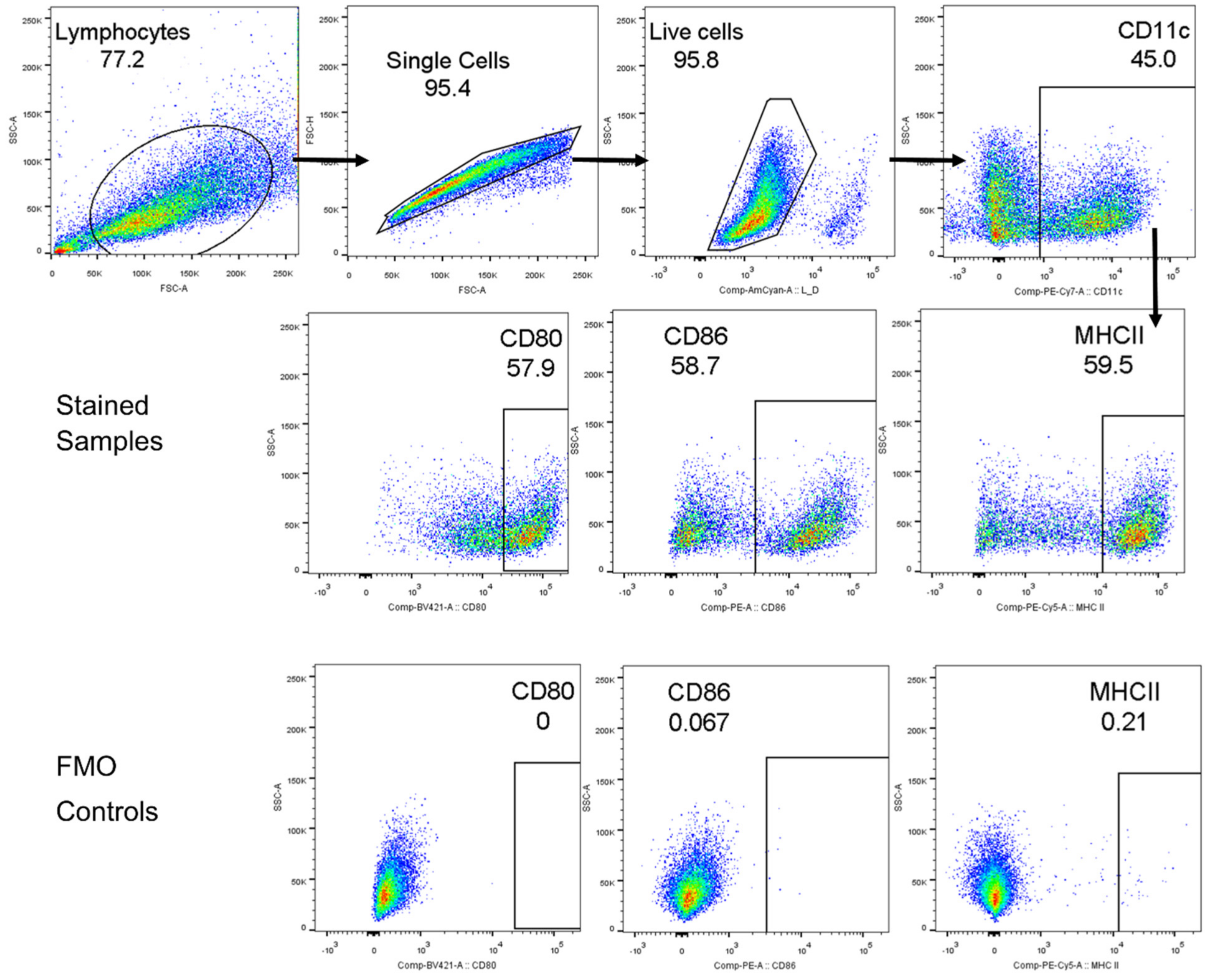

Figure S3. The gating strategy used for the DC ex-vivo activation assay. Hierarchical gating was used to identify the total cell population followed by single live cells the CD11C+ DC. Out of the CD11C population, the expression of DC cell activation markers was determined based on the fluorescence minus one (FMO) controls.

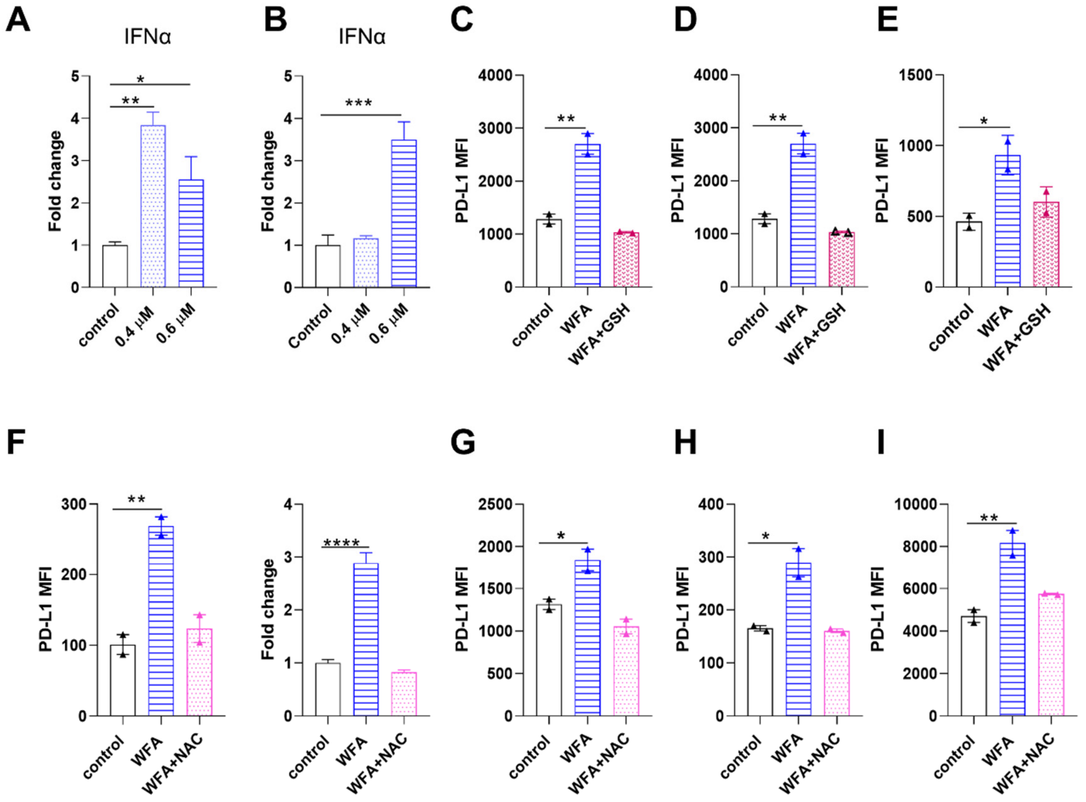

Figure S4. WFA treatment increased the mRNA levels of IFNα in (**A**) LLC and (**B**) H1650 cell lines. ROS is the main regulator of WFA-induced PD-L1 upregulation. Flow cytometry analysis shows that NAC (5 mM) or GSH (5 mM) abrogated WFA-mediated PD-L1 upregulation in (**C**) LLC, (**D**) H1650 and (**E**) A549 (**F**) MC-38, (**G**) HCT-116 colorectal cancer cell lines, (**H**) 4T1, and (**I**) MDA-MB-231 breast cancer cell lines. All cells were treated with 0.6 µM WFA except A549, 4t1 and MDA-MB-231 cells which were treated with 4 µM, 1 µM, 2 µM WFA, respectively. (**F**) qRT-PCR shows that NAC abrogates WFA-mediated PD-L1 upregulation in MC-38 cells. PCR experiments were repeated twice, and one representative experiment is shown as mean ± SEM of 3 technical replicates. Flow cytometry experiments were performed twice and the mean ± SEM of two representative experiments is shown. Treatments were compared to the control using one-way ANOVA and Fisher LSD post hoc test. * $p < 0.05$, ** $p < 0.01$, *** $p < 0.001$, **** $p < 0.0001$.

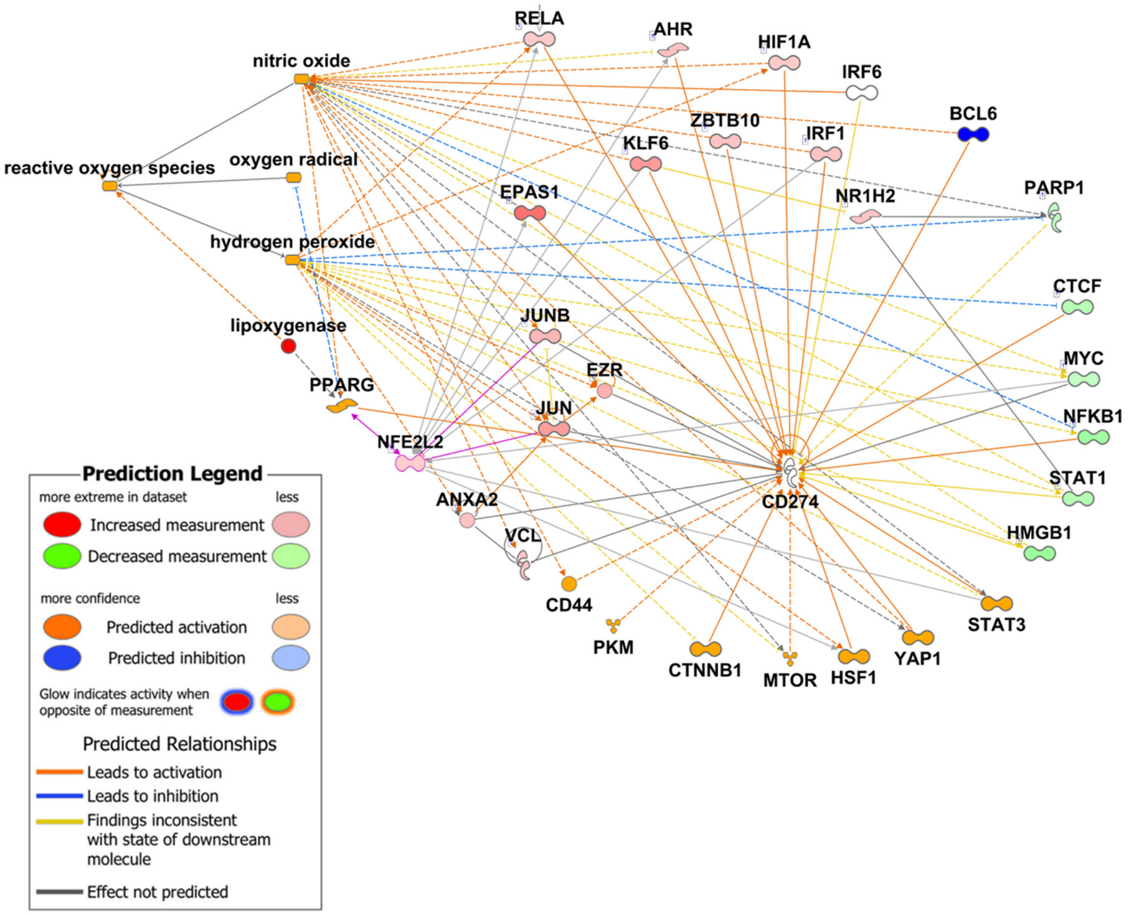

Figure S5. A gene interaction network between the PDL1 signaling pathway and reactive oxygen species (nitric oxide, oxygen radical, hydrogen peroxide, and lipoxygenase) signaling was generated using known connections obtained from the Ingenuity Pathway Analysis database. Fold change data from MCF-7 breast cancer cells treated with withaferin A (700nM for 72hr) was obtained from NCBI Gene Omnibus Expression Database (Series GSE53049) and overlaid onto the network to yield predictions of activation/inhibition in the IPA software.

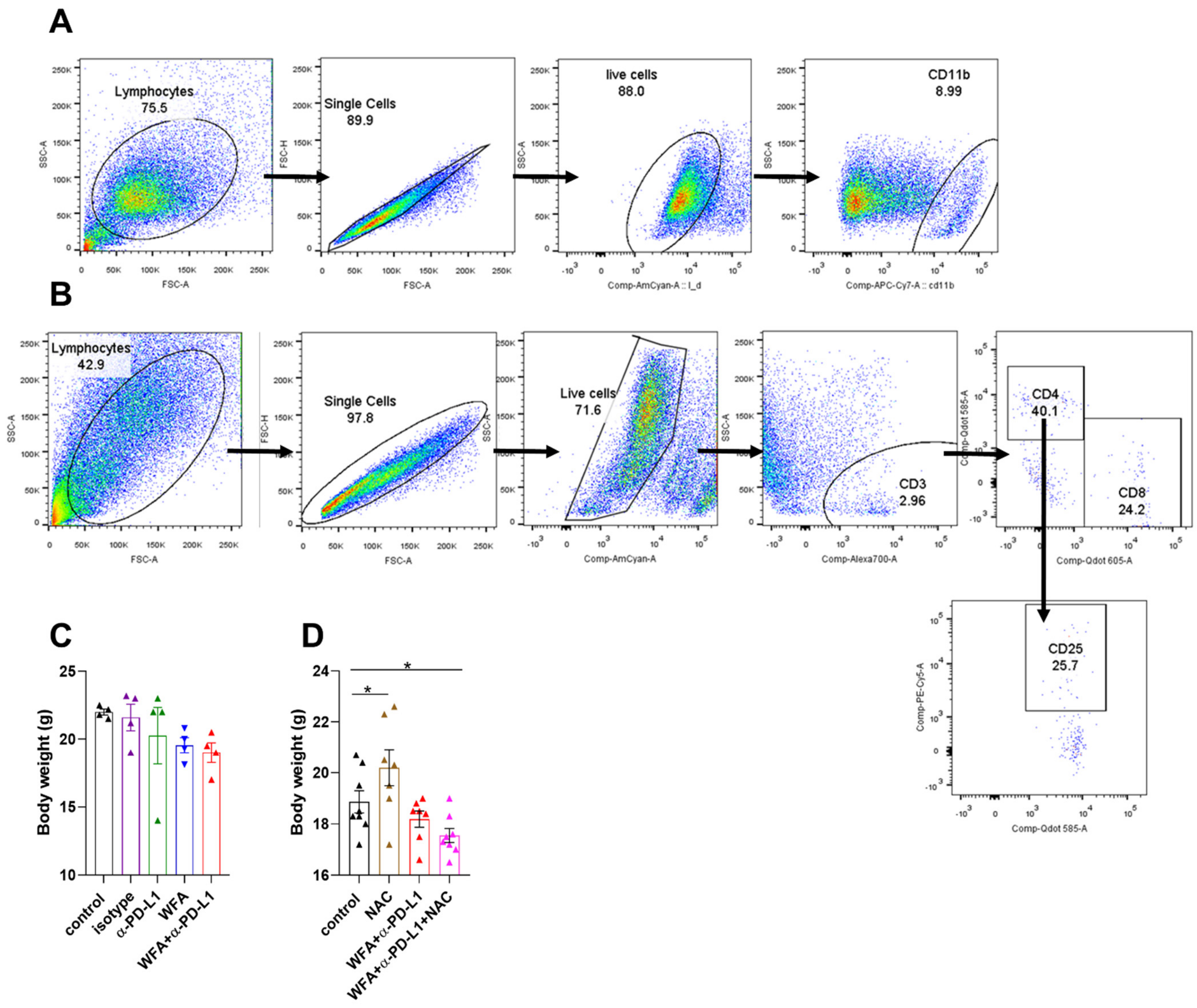

Figure S6. The gating strategy used to determine tumor immune infiltration in LLC tumors and spleens. Hierarchical gating was used to identify the total cell population followed by single live cells then (**A**) the CD11b+ myeloid cells. Out of the CD11b population, we gated the monocytic CD11b+ Ly6C+ and CD11b+ Ly6G+ granulocytic MDSCs. (**B**) For the T-cell panel, the total CD-3 population was gated out of the live single cells. Consequently, CD4, CD25+CD4+ T-regs and CD8 population were gated out of CD-3 cells based on the FMO controls. (**C**,**D**) Mice body weights were measured at the endpoint of the experiment and represented as mean ± SEM. The means were compared to the vehicle control using Fisher LSD post-hoc test. * $p < 0.05$.

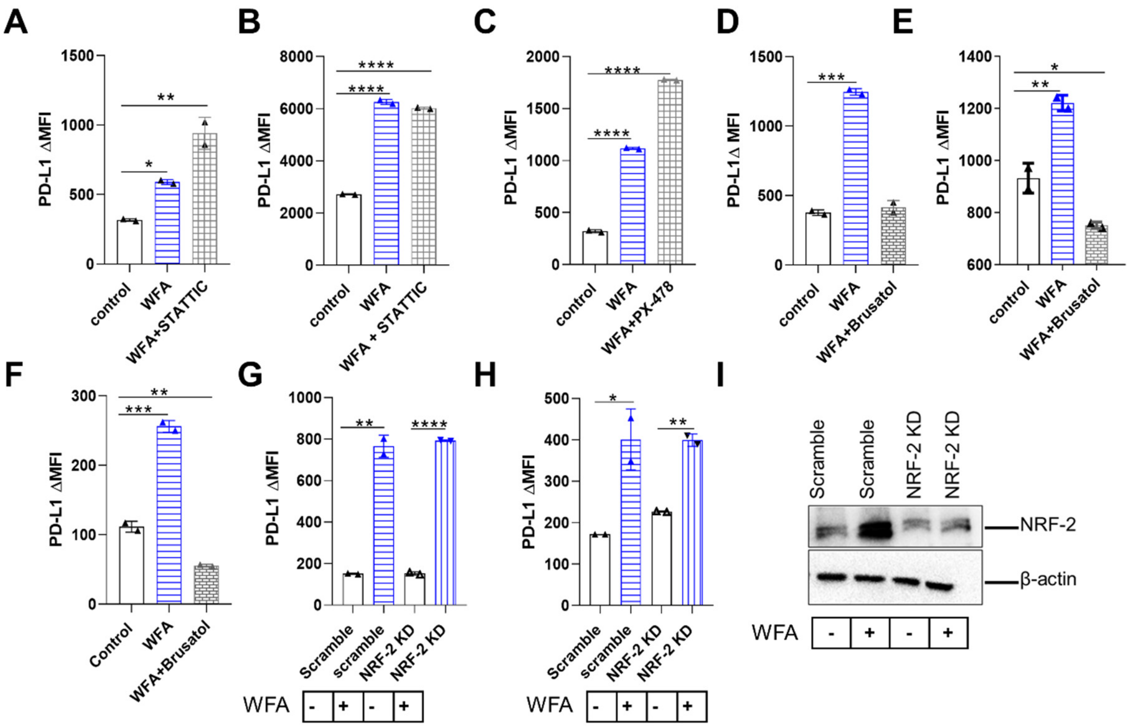

Figure S7. Investigating the downstream molecular regulators that may be involved in WFA-mediated PD-L1 upregulation. LLC (**A**) and H1650 (**B**) were treated with either WFA or WFA+STATTIC for twenty-four hours then PD-L1 expression was measured using flow cytometry. (**C**) LLC cells were treated with WFA or WFA+PX-478 then collected for PD-L1 measurement using flow cytometry. LLC (**D**), H1650 (**E**), and A540 cells (**F**) were treated with WFA or WFA+Brusatol then collected for PD-L1 measurement using flow cytometry. LLC (**G**) or H1650 (**H**) were transfected with a NRF-2 or scramble siRNA then treated with WFA for twenty-four hours to measure PD-L1 levels by flow cytometry. (**I**) NRF-2 knockdown was confirmed by western blotting of scramble and siRNA-transfected cells. Flow cytometry experiments were repeated at least twice and the means ± SEM are shown in the figures and compared using one-way ANOVA and Fisher LSD post hoc test. * $p < 0.05$, ** $p < 0.01$, *** $p < 0.001$, **** $p < 0.0001$.
